# A decade of global volcanic SO_2_ emissions measured from space

**DOI:** 10.1038/srep44095

**Published:** 2017-03-09

**Authors:** S. A. Carn, V. E. Fioletov, C. A. McLinden, C. Li, N. A. Krotkov

**Affiliations:** 1 Department of Geological and Mining Engineering and Sciences, Michigan Technological University, Houghton, MI 49931, USA; 2 Air Quality Research Division, Environment and Climate Change Canada, Toronto, ON, Canada; 3 Earth System Science Interdisciplinary Center, University of Maryland, College Park, MD, USA; 4 Atmospheric Chemistry and Dynamics Laboratory, Code 614, NASA Goddard Space Flight Center, Greenbelt, MD 20771, USA

## Abstract

The global flux of sulfur dioxide (SO_2_) emitted by passive volcanic degassing is a key parameter that constrains the fluxes of other volcanic gases (including carbon dioxide, CO_2_) and toxic trace metals (e.g., mercury). It is also a required input for atmospheric chemistry and climate models, since it impacts the tropospheric burden of sulfate aerosol, a major climate-forcing species. Despite its significance, an inventory of passive volcanic degassing is very difficult to produce, due largely to the patchy spatial and temporal coverage of ground-based SO_2_ measurements. We report here the first volcanic SO_2_ emissions inventory derived from global, coincident satellite measurements, made by the Ozone Monitoring Instrument (OMI) on NASA’s Aura satellite in 2005–2015. The OMI measurements permit estimation of SO_2_ emissions from over 90 volcanoes, including new constraints on fluxes from Indonesia, Papua New Guinea, the Aleutian Islands, the Kuril Islands and Kamchatka. On average over the past decade, the volcanic SO_2_ sources consistently detected from space have discharged a total of ~63 kt/day SO_2_ during passive degassing, or ~23 ± 2 Tg/yr. We find that ~30% of the sources show significant decadal trends in SO_2_ emissions, with positive trends observed at multiple volcanoes in some regions including Vanuatu, southern Japan, Peru and Chile.

Accurate inventories of the current spatial and temporal distribution of volcanic gas emissions to the atmosphere are required for numerous applications, ranging from baseline volcano monitoring to assessment of the impacts of volcanic degassing on the broader Earth system^1^. Sulfur species, principally sulfur dioxide (SO_2_), are of most interest due to the ease of SO_2_ measurement via ground- and satellite-based remote sensing^2^,^3^ and their key role in the processes responsible for volcanic impacts on the environment, health, atmospheric chemistry and climate^4^,^5^,^6^,^7^. Recent advances in satellite remote sensing techniques have greatly improved constraints on the eruptive flux of SO_2_ (and several other volatile species) from volcanoes^3^,^8^,^9^,^10^, but the non-eruptive or passive volcanic degassing flux of SO_2_ (hereafter, PVF) remains poorly constrained. In addition to its relevance for impact assessment, an accurate global volcanic SO_2_ emissions inventory permits estimation of the volcanic output of other climate-relevant gas species and toxic trace metals (e.g., CO_2_ and mercury^11^,^12^), and the identification of potential targets for ground-based gas sampling to measure the complete chemical and isotopic composition of volcanic gases. The most widely used existing volcanic SO_2_ emissions inventory^13^ is now several decades old, but its enduring popularity reflects the high demand for global volcanic SO_2_ flux data.

Producing a database that faithfully reflects the contemporaneous PVF is a challenge due to the generally poor temporal and spatial coverage of ground-based volcanic gas measurements, which are often conducted on a campaign-style basis and/or during periods of heightened unrest^14^,^15^,^16^ and are hence unlikely to accurately represent long-term average degassing rates. Although the geographic extent and frequency of ground-based volcanic SO_2_ measurements is increasing^17^, they remain sparse in many highly active volcanic regions such as Indonesia, Papua New Guinea, Vanuatu, the Aleutian Islands, the Kuril Islands and Kamchatka, and will be a formidable challenge in some very remote regions (e.g., the South Sandwich Islands, southern Atlantic Ocean). As a solution to this problem, we report here a new satellite-based volcanic SO_2_ emissions inventory, based on more than a decade of measurements by the Ozone Monitoring Instrument (OMI) on NASA’s Aura satellite^18^, which is global in scope and provides estimates of the PVF from all of the strongest contemporary volcanic SO_2_ sources. This new database, the first volcanic SO_2_ emissions inventory to be derived from global, coincident measurements (rather than by collation of ground-based data widely distributed in space and time), benefits from several advantages of polar-orbiting satellite measurements, including global coverage and the use of a single, well-characterized sensor to detect and quantify all SO_2_ sources over the course of a long-term (multi-decadal) satellite mission. The use of a single instrument permits relatively straightforward reprocessing of archived data as SO_2_ retrieval algorithms improve, offering increasing sensitivity to volcanic SO_2_^19^. Furthermore, unlike many spectroscopic instruments used for ground-based SO_2_ measurements, satellite instruments such as OMI are also subject to intensive calibration and validation^20^.

Although satellites have been used to measure eruptive SO_2_ emissions for several decades^3^,^8^,^21^,^22^, their use for quantification of passive volcanic degassing is relatively recent and concurrent with the advent of sufficiently sensitive space-borne instruments, such as OMI^23^,^24^,^25^. Previous application of OMI SO_2_ data to detection of non-eruptive volcanic degassing has focused on the stronger SO_2_ sources, detectable from space on a near-daily basis^3^,^16^,^25^. As recently demonstrated^26^,^27^,^28^,^29^,^30^, with specialized data processing techniques it is possible to enhance the sensitivity of ultraviolet (UV) satellite SO_2_ measurements to enable detection of persistent anthropogenic SO_2_ sources emitting on the order of 30 kilotons/year (kt/yr; equivalent to ~80 tons/day [t/d]), with the detection limit expected to be even lower for SO_2_ sources located at high elevation (including many volcanoes). Here, we present a new global volcanic SO_2_ emissions inventory derived from application of these techniques to more than a decade of OMI observations (2005–2015), which represents a timely replacement for existing databases^13^,^31^. We also compare the satellite-based SO_2_ fluxes to a recent compilation of independent ground-based measurements^31^, and other sources, and examine the global distribution of volcanic SO_2_ fluxes to reveal regional- and arc-scale trends in volcanic degassing.

## Data and Methods

**Figure 1:**
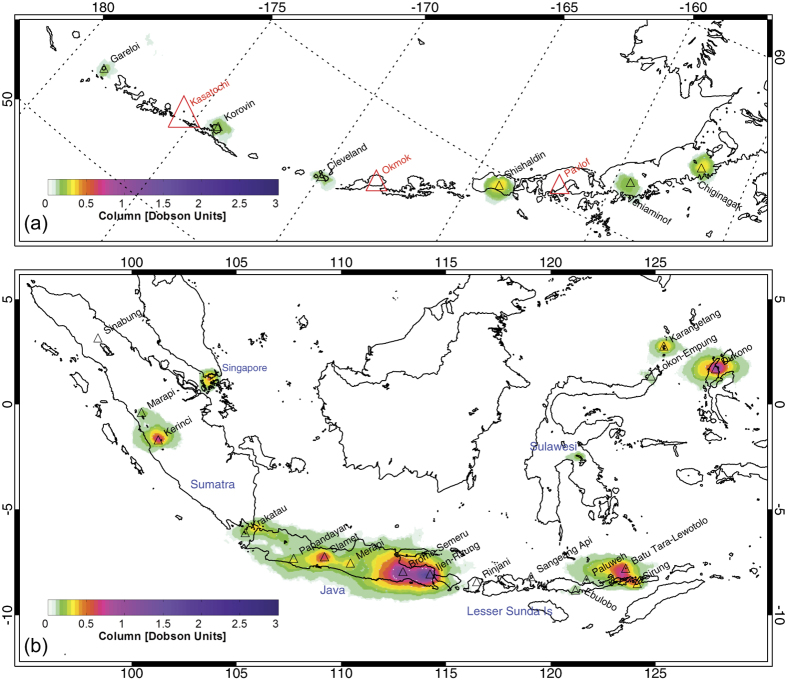
Mean SO_2_ columns (in Dobson Units [DU]; 1 DU = 2.69 × 10^16^ molecules cm^−2^) for 2005–2007 over (**a**) the Aleutian Islands (USA) and (**b**) Indonesia. The volcanic SO_2_ sources (including paired sources) are labeled. The Aleutian map also shows locations of explosive eruptions since 2005 (*red triangles*), with symbol size proportional to total SO_2_ emission^3^,^10^. The Indonesian map also shows anthropogenic SO_2_ sources in Singapore and central Sulawesi, but does not show volcanic SO_2_ emissions from Sinabung, Rinjani and Sangeang Api, which first appeared after 2007. Maps were generated using Interactive Data Language (IDL) version 8.5.1 (http://www.harrisgeospatial.com/).

**Figure 2:**
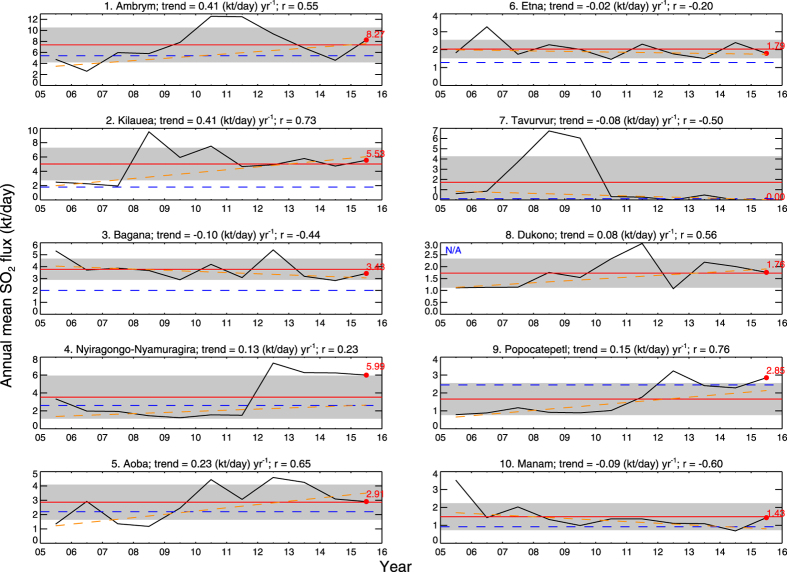
OMI-derived annual mean SO_2_ fluxes in 2005–2015 for the ten strongest volcanic SO_2_ sources (including paired sources) in the inventory. Plots are titled with the volcanic source name and rank, and the trend (slope) and linear correlation coefficient (*r*) of an error-weighted linear regression fit of the annual mean SO_2_ fluxes. Each plot shows the annual mean SO_2_ fluxes (*solid black line*), mean SO_2_ flux in 2015 (*labeled red dot*), linear regression trend line (*dashed orange line*), decadal mean SO_2_ flux (*horizontal red line*), ±1 standard deviation of the decadal mean SO_2_ flux (*gray band*), and an independent estimate of SO_2_ flux (*horizontal dashed blue line*) from a recent compilation^13^ or another source. Here, SO_2_ flux data for Etna and Popocatepetl are from refs 58 and 59, respectively. If no independent measurements are available, the plot is labeled with ‘N/A’. See Supplementary Figures (Figs S9–S16) for similar plots for all other sources.

Volcanic SO_2_ emissions were estimated using a new operational OMI planetary boundary layer (PBL) SO_2_ column dataset produced using a principal component analysis (PCA) algorithm^32^. A detailed description of the techniques used to identify SO_2_ sources (both anthropogenic and volcanic) and calculate emissions is given in ref. 29, and is briefly summarized here. The OMI PCA SO_2_ data used in the analysis were restricted to ‘clear sky’ conditions by including only those OMI pixels with a cloud radiance fraction below 20%; solar zenith angles were also restricted to <70° to reduce noise at high latitudes. In addition, all pixels affected by the OMI row anomaly data gap since 2007 (see: http://www.knmi.nl/omi/research/product/rowanomaly-background.php) were excluded. After pixel screening, an OMI pixel averaging or oversampling procedure^26^,^27^ is used to resolve potential locations of SO_2_ emissions and produce global maps similar to those shown in Fig. 1. To further enhance the SO_2_ signal and identify sources, a wind rotation technique is applied to align all the OMI SO_2_ observations for each source along the same wind vector^30^, and then SO_2_ emissions are estimated by fitting an exponentially modified Gaussian function to the OMI data^33^. The variable altitude of passive volcanic SO_2_ plumes is accounted for by applying an air mass factor (AMF) correction to the OMI PBL SO_2_ columns based on volcano altitude. To calculate accurate estimates of the SO_2_ PVF, the effects of volcanic eruptions generating transient, large SO_2_ column amounts are removed by applying a threshold SO_2_ column amount of 5–15 Dobson Units (DU) to the OMI SO_2_ data. This threshold was selected based on typical SO_2_ column amounts measured by OMI in passive and eruptive volcanic plumes. However, we note that at some volcanoes it may be impossible to completely separate passive (i.e., involving no coincident eruption of magma) from eruptive SO_2_ emissions, or even to establish which mode of degassing dominates at any given time. This is particularly problematic at volcanoes undergoing lava dome extrusion (e.g., Merapi, Indonesia; Soufriere Hills, Montserrat) or persistent Vulcanian or Strombolian activity (e.g., Stromboli, Italy; Fuego, Guatemala; Sakura-jima, Japan; Yasur, Vanuatu). Hence, while we believe that passive SO_2_ degassing is the dominant process responsible for the emissions reported here, a contribution from eruptive degassing is inevitable at some volcanoes, as is the case for previous SO_2_ emissions inventories^13^,^31^. Total uncertainties (including contributions from AMF, SO_2_ mass, SO_2_ lifetime, and wind speed uncertainty) on annual SO_2_ flux estimates are 55% and >67% for sources emitting more than 100 kt/yr and under 50 kt/yr, respectively^29^. Some of the largest individual sources of error are systematic and hence will introduce a bias in absolute SO_2_ flux values but will not affect relative inter-annual flux variability (Fig. 2).

For the inventory presented here, volcanic SO_2_ sources were identified based on 3-year averages of OMI data for 2005–2007, 2008–2010 and 2011–2014, then annual emissions were calculated for each source for the entire 11-year period studied (2005–2015). Note that the aforementioned 30 kt/yr (~80 t/d) detection limit was determined based on OMI observations of power plant SO_2_ emissions in the eastern USA^28^, which are typically confined to the PBL. The higher altitude of volcanic SO_2_ plumes translates into a higher AMF (greater sensitivity), which reduces the detection limit to values as low as ~6 kt/yr (~16 t/d). The detection limit will be lowest for low-latitude volcanoes, which benefit from more satellite observations under optimal conditions (e.g., low solar zenith angles). To assess the presence of significant decadal trends in the SO_2_ emissions, we applied a weighted linear regression fit to the annual SO_2_ emissions for each source, using the 1σ emission uncertainties (Supplementary Table S1) to weight the data, to derive a trend and linear correlation coefficient (*r*). Although it is possible to use satellite data to estimate SO_2_ fluxes on much shorter timescales for strong sources^9^,^25^, the focus here is on long-term average emissions and trends rather than short-term variations. Future updates to the volcanic SO_2_ emissions inventory will benefit from the recent release of new OMI PCA SO_2_ products tailored to the variable injection height of volcanic plumes^19^, which should further reduce the uncertainties.

## Results and Discussion

A total of 91 persistently degassing volcanic SO_2_ sources have been detected in OMI measurements between 2005 and 2015 (Table 1; Supplementary Fig. S1). However, some of the detected SO_2_ signals originate from paired sources (see below), so the actual number of volcanoes contributing to the detected SO_2_ emissions is probably at least 100. For comparison, the Andres and Kasgnoc (1998) inventory^13^ includes 49 continuously emitting sources. Since 3-year averages of OMI SO_2_ data were used to identify the sources, the main criterion for detection is persistent emissions on that timescale. Hence it is possible that volcanoes exhibiting shorter-duration episodes of passive degassing may elude detection, but may subsequently be identified in more detailed analysis of shorter time periods. Table 1 lists the volcanic SO_2_ sources, ranked according to their mean SO_2_ flux for the entire 11-year period analyzed. Maps of the volcanic SO_2_ sources are shown in Fig. 1 and Supplementary Figs S1–S8. Figure 2 and Supplementary Figures (Supplementary Figs S9–S16) show the variation in annual mean SO_2_ fluxes at each source, with correlative ground-based SO_2_ measurements if available, and the trends and linear correlation coefficients (*r*) derived from a weighted linear regression of the annual emissions. The complete dataset, including annual emissions for each volcano, is provided in a Supplementary Table (Supplementary Table S1).

One of the disadvantages of UV satellite measurements is low spatial resolution, and as a result SO_2_ emissions from clustered degassing volcanoes (within ~50 km) cannot be distinguished. Hence, some SO_2_ emissions in the inventory are attributed to paired sources (e.g., Fig. 2), such as Nyiragongo-Nyamuragira (DR Congo), Bromo-Semeru (East Java, Indonesia) and Batu Tara – Lewotolo (Lesser Sunda Islands, Indonesia). Kamchatka (Russia) is another region where assignment of SO_2_ emissions to specific volcanoes can be problematic (e.g., Mutnovsky – Gorely). Emissions reported for Chikurachki in the northern Kuril Islands may include a contribution from Ebeko (Table 1), where SO_2_ emissions of ~100 t/d have been reported^34^. Resolving these merged SO_2_ sources will require further field-based measurements in some regions, or the use of satellite data with higher spatial resolution^35^.

Notwithstanding some drawbacks, the strength of a satellite-derived emissions inventory is the global coverage. Most of the dominant sources (e.g., Ambrym, Kilauea, Bagana, Etna) are well established from prior measurements^14^,^16^,^36^,^37^. However, the OMI measurements (Table 1; Fig. 2) reveal, in some cases for the first time, significant, persistent SO_2_ degassing at remote volcanoes in the South Sandwich Islands (Michael and Montagu), the Kuriles (Ketoi, Kudriavy), the Aleutians (Gareloi, Korovin), Indonesia (e.g., Dukono, Batu Tara - Lewotolo, Sirung, Ebulobo), and the southwest Pacific (e.g., Tofua, Tinakula). Gas emissions from Erebus (Antarctica) are also detected from space for the first time (Table 1; Supplementary Fig. S16). The OMI database thus provides what is the first truly global picture of contemporary volcanic SO_2_ degassing, including sources where acquisition of frequent ground-based data will remain highly challenging.

Weighted linear regression reveals a range of temporal trends in the SO_2_ fluxes (Fig. 2; Supplementary Figs S9–S16). We acknowledge that a simple linear trend may not be applicable to many of the volcanic SO_2_ sources (indicated by a low correlation coefficient, −0.5 ≤ *r* ≤ 0.5; Fig. 2; Supplementary Figs S9–S16), but a detailed exploration of the trends in SO_2_ emissions at each volcano is beyond the scope of this study. Nevertheless, the SO_2_ data for some sources clearly indicate a long-term decline in SO_2_ discharge (e.g., Miyakejima, Manam, Soufriere Hills; Fig. 2, Supplementary Fig. S9). A weak or insignificant trend in SO_2_ emissions likely reflects relatively stable emissions (e.g., Bagana, Etna; Fig. 2), or more pulsatory degassing (e.g., Tavurvur, Anatahan, Huila; Fig. 2, Supplementary Figs S9 and S10); the latter could reflect cycles of magma intrusion followed by protracted gas release. Three of the top four sources feature active basaltic lava lakes (Ambrym, Kilauea and Nyiragongo-Nyamuragira), and in these cases the peak SO_2_ discharge can be clearly linked to the establishment of new and/or larger lava lakes (e.g., at Kilauea in 2008^38^ and Nyamuragira in 2012^35^). The significance of the observed trends in SO_2_ emissions is discussed further below.

**Figure 3:**
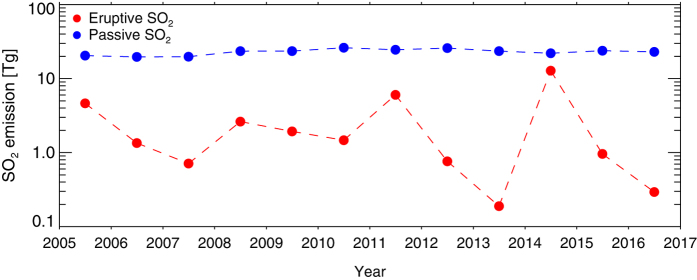
Total, global eruptive and passive volcanic SO_2_ emissions (in Tg; 1 Tg = 10^12^ g) in 2004–2016. Eruptive emissions are derived from [10] and recent updates; passive emissions are from the inventory described here. Passive volcanic SO_2_ emissions in 2016 are assumed to continue at the mean annual rate observed in 2005–2015.

In summing the SO_2_ emissions from all detected sources, we find that the total annual SO_2_ PVF is remarkably stable at 23.0 ± 2.3 Tg/yr (the highest annual total in the past decade was ~26 Tg in 2010). Andres and Kasgnoc (1998)^13^ estimated a total non-eruptive volcanic SO_2_ flux of ~12 Tg/yr for the 1970–1997 period (including a power-law extrapolation to estimate the contribution from unmeasured volcanoes); our higher estimate reflects the inclusion of more strong sources emitting >1000 t/d SO_2_ (Table 1). A comparison with eruptive SO_2_ fluxes^3^,^10^ confirms the common assumption that the SO_2_ PVF is typically around an order of magnitude larger (Fig. 3), except during years with major SO_2_-rich eruptions such as at Bárðarbunga-Holuhraun (Iceland) in 2014^39^. The average total SO_2_ PVF from all detectable sources is ~63 kt/day (2005–2015 mean; Table 1), which is broadly commensurate with a global SO_2_ PVF of ~50.6 kt/day estimated by ref. 31 using a sparser dataset. Fluxes of SO_2_ during large eruptions (e.g., Holuhraun^39^) can greatly exceed the total PVF on short timescales.

The new volcanic SO_2_ emissions inventory includes numerous previously unquantified sources. Based on SO_2_ data reported in the literature (and we acknowledge that a substantial amount of SO_2_ emissions data collected by volcano observatories may not be published), we find that 36 of the 91 sources (i.e., ~40%) have no previously reported SO_2_ flux. The most prominent of these is Dukono (Halmahera, Indonesia), ranked 8^th^ in our inventory (Table 1; Figs 1 and 2), but many of the stronger sources have relatively few SO_2_ flux determinations. Based on recent compilations^31^, 38 volcanoes (i.e., ~68% of the 56 volcanoes with prior measurements) have reported SO_2_ fluxes within the 1σ fitting uncertainty of the OMI-derived fluxes. For ~41% of the sources with prior measurements, the OMI-derived SO_2_ flux exceeds the independent estimate by at least 20%, and for ~36% the reverse is true (Fig. 2; Supplementary Figs S9–S16), whilst for the remainder (e.g., Ulawun, San Cristobal, Satsuma-Iwojima, Masaya, Fuego; Supplementary Figs S9–S16) the satellite- and ground-based SO_2_ emission rates show excellent agreement (to within 20%). Nonetheless, it is notable that the OMI-derived SO_2_ fluxes for most of the strongest sources are higher than previous estimates (Table 1; Fig. 2). For several sources (e.g., Ambrym, Bagana, Aoba, Manam) we believe that this is real and a result of infrequent prior measurements at these very active volcanoes coupled with significant variability in SO_2_ emissions. Furthermore, at Kilauea, where a significant discrepancy is observed (Fig. 2), it has recently been shown^37^ that ground-based techniques can underestimate SO_2_ emissions by a factor of 2 or more in dense plumes. However, with the exception of the high-flux volcanoes, we observe no significant high or low bias in the OMI-derived SO_2_ fluxes, but more detailed validation of the derived SO_2_ emissions is certainly required.

In addition to Dukono, the new database sheds considerable light on the SO_2_ flux from other Indonesian volcanoes (Fig. 1), which is noteworthy given the generally poor constraints on volcanic emissions in the archipelago^40^,^41^. Dukono (Halmahera) is the strongest volcanic SO_2_ source with no prior constraints on its SO_2_ flux (Figs 1 and 2). The SO_2_ signal in the Sunda Strait near Krakatau volcano (Fig. 1) was previously assigned to the Suralaya power plant in Cilegon, West Java^29^, but we now assume this to be dominated by volcanic emissions from Krakatau (Table 1). SO_2_ emissions of 190 ± 40 t/d were reported at Krakatau in 2014^42^, well above the satellite detection limit, so if this is a sustained SO_2_ flux then it seems likely that most of the detected SO_2_ is volcanic. The OMI-derived SO_2_ flux for Krakatau is 303 ± 252 t/d (Table 1), i.e., within the range of ground-based measurements^42^. Degassing from Papandayan (West Java^40^) may also be detected in the OMI data (Fig. 1), although it is difficult to isolate from the larger SO_2_ signal associated with Slamet and hence is not treated as a separate source here. As noted earlier, several Indonesian volcanoes in East Java and the Lesser Sunda Islands are difficult to resolve using the OMI measurements, thus the reported emissions for Bromo and Semeru, Raung and Ijen, and Batu Tara and Lewotolo represent aggregated fluxes (Table 1). Ground-based SO_2_ measurements in Indonesia are also increasing in frequency and coverage^40^,^41^,^42^,^43^,^44^. The OMI-derived average SO_2_ flux from Bromo-Semeru (775 ± 298 t/d; Table 1) is higher than combined ground-based estimates for these volcanoes (~200 t/d^41^,^44^), but the ground-based campaigns only cover a few days of degassing. It is also possible that the satellite measurements are more effective than ground-based techniques at constraining SO_2_ flux at volcanoes that exhibit transitions from purely passive degassing to degassing via Vulcanian explosions (e.g., Semeru), due to the difficulty of measuring SO_2_ in proximal ash-laden plumes^44^.

Another notable feature apparent in the map of Indonesian SO_2_ sources is that some regions show lower emissions or an absence of subaerial SO_2_ degassing, despite the presence of numerous Holocene volcanoes; e.g., southern Sumatra and the western Lesser Sunda Islands (Fig. 1). It is perhaps no coincidence that the latter region is the location of several volcanoes responsible for large SO_2_-rich explosive eruptions (linked to significant climate impacts^5^) including Agung (1963)^45^, Samalas (1257)^46^ and Tambora (1815)^47^. The identification of such degassing gaps, where stored gas may be accumulating in magma reservoirs rather than being released to the atmosphere, could assist hazard mitigation and identification of potential sites of future explosive eruptions. The mutually exclusive relationship between strong subaerial SO_2_ degassing and large explosive eruptions during the past decade is also apparent in the Aleutian Islands (Fig. 1).

Further corroboration of the OMI-derived SO_2_ emissions is possible based on data collected at Japanese volcanoes. A recent assessment^48^ showed that 94% of the total volcanic SO_2_ flux in Japan originates from 6 volcanoes: Tokachi, Asama, Aso, Sakurajima, Satsuma-Iwojima, and Suwanosejima; plus Mijake-jima after 2000. A total of 17 degassing volcanoes are documented in Japan^48^. OMI is able to detect all seven of the strongest sources (Table 1), yielding a time-averaged total SO_2_ flux for Japan of 1.73 Tg/yr in 2005–2015, which is commensurate with a total SO_2_ flux of 2.2 Tg/yr (including the intense degassing from Miyake-jima after 2000, which continues to subside) or 1.4 Tg/yr pre-2000 based on ground-based data^48^. Thus the OMI measurements represent an accurate estimate of total volcanic SO_2_ emissions from Japan during the ongoing waning phase of Miyake-jima’s degassing activity.

**Figure 4:**
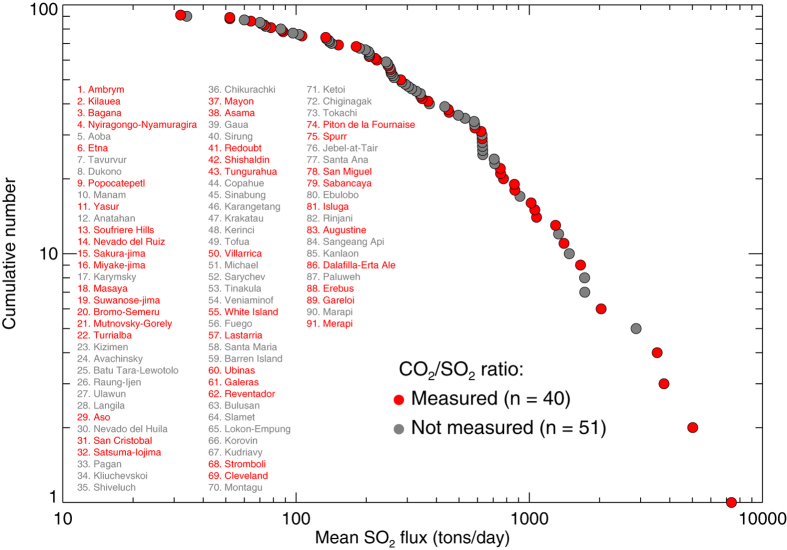
Cumulative frequency – SO_2_ flux plot for all volcanic SO_2_ sources detected by OMI. Symbol color indicates whether the CO_2_/SO_2_ ratio of the volcanic gases has been measured as of October 2016. Information on availability of CO_2_/SO_2_ ratios is from E. Hauri (DCO-DECADE, pers. comm.).

Examination of the frequency-flux relationship of volcanic SO_2_ fluxes in Japan reveals that they do not fit a power law distribution^48^, as had been previously suggested for the global flux distribution^49^. A frequency-flux plot for the OMI-derived SO_2_ emissions confirms that the global volcanic SO_2_ sources also do not follow a power law distribution (Fig. 4). We also find a clear ‘roll-off’ of the distribution at an SO_2_ flux of ~500–600 t/d, remarkably similar to that found in the ground-based Japanese SO_2_ flux data^48^. This important result shows that the distribution of volcanic SO_2_ emissions on the scale of individual arcs can indeed mimic the global distribution, provided that large flux datasets are available from a range of source strengths (i.e., including very strong emitters such as Miyake-jima). It also indicates that the global volcanic SO_2_ flux is dominated by the ~30 largest sources (Table 1; Fig. 4), and quantifying the flux from these volcanoes would provide a good estimate of the global SO_2_ flux (in our database the 30 strongest sources emit ~80% of the total flux).

### Arc-scale trends in volcanic degassing

**Figure 5:**
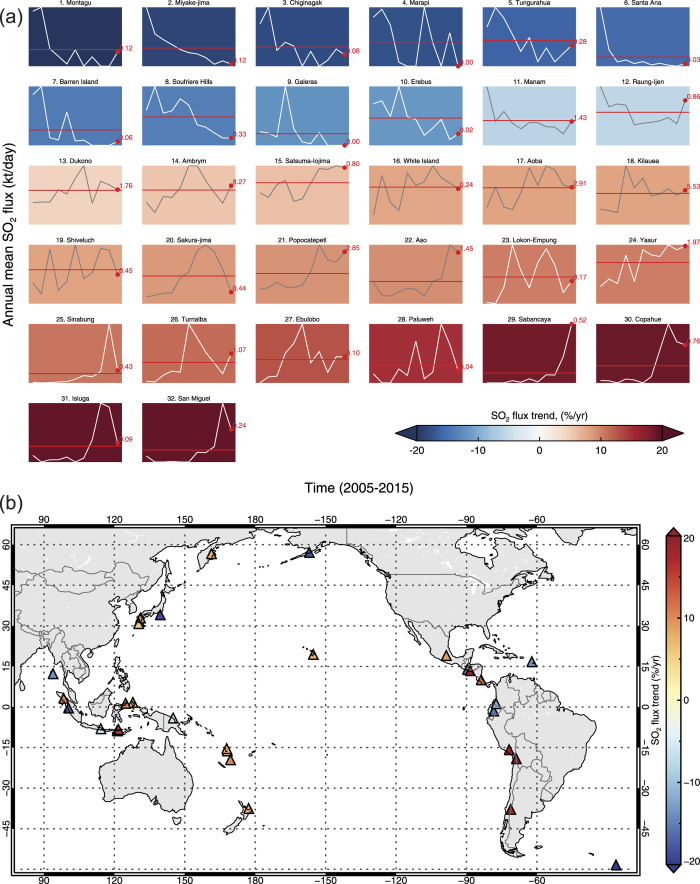
(**a**) Decadal trends in SO_2_ emissions measured at 32 volcanic SO_2_ sources showing a significant linear correlation coefficient from a weighted linear regression fit (*r* ≤ −0.5 or *r* ≤ 0.5). Plots are ranked in order of calculated SO_2_ flux trend (i.e., the slope of the linear fit) from negative to positive values. Hence, cold and warm colors indicate sources showing a significant reduction or increase in SO_2_ emissions over the 11-year period of measurements, respectively. Each individual plot shows the annual mean SO_2_ fluxes for 2005–2015 (*white-gray line*), the decadal mean SO_2_ flux (*red line*) and the annual mean SO_2_ flux in 2015 (*labeled red dot*) for each source; axis labels are omitted for clarity. The vertical scale on each plot extends from zero to the maximum measured SO_2_ flux. For more detailed time-series plots, see Fig. 2 and Supplementary Figures (Figs S9–S16); (**b**) Location map of the 32 volcanic SO_2_ sources, colored based on SO_2_ flux trend in 2005–2015 (also see Supplementary Figure S1). Map generated using Interactive Data Language (IDL) version 8.5.1 (http://www.harrisgeospatial.com/).

Another significant application of the global satellite SO_2_ measurements is the potential for detection of arc-scale trends in gas flux. Global, consistent SO_2_ measurements such as the OMI-derived database presented here pave the way to new insights into arc-scale volcanic processes, including correlations between volcanic SO_2_ emissions and other geophysical parameters such as arc length and subduction rate, since they provide a synoptic perspective on degassing that is not easily obtained from other techniques. The application of pattern recognition techniques to global SO_2_ emissions data, such as the example in Fig. 5 (also see Supplementary Fig. S17), will permit an epidemiological approach whereby analogous degassing patterns may be identified at similar volcanic systems on regional or global scales. Interpretation of SO_2_ data at individual volcanic systems can be ambiguous^50^, but analysis of arc-scale SO_2_ measurements potentially allows the identification of correlated trends at multiple volcanoes that can be more confidently ascribed to similar volcanic processes.

The recent status of SO_2_ emissions at the detected volcanic sources can be straightforwardly assessed by comparing the most recently measured annual mean SO_2_ flux (for 2015) with the decadal mean flux (Supplementary Figs S1 and S17). This simple metric shows some notable arc-scale consistency in several regions; for example, all the detected volcanic SO_2_ sources in Peru and Chile (Isluga, Villarrica, Lastarria, Ubinas, Copahue, and Sabancaya) have measured emissions in 2015 that are above the long-term average (Supplementary Figs S1 and S17). In southern Peru, both Ubinas and Sabancaya show particularly anomalous SO_2_ emissions in 2015 (Table 1; Supplementary Figs S1 and S17), suggesting that these volcanoes are currently in a period of elevated activity. In contrast, the volcanoes of Papua New Guinea (Tavurvur, Langila, Bagana, Manam, and Ulawun) all show recent SO_2_ emissions close to or below the decadal mean (Supplementary Figs S1 and S17).

A more rigorous evaluation of trends in SO_2_ emissions must be restricted to those sources with annual SO_2_ emissions showing a significant positive or negative linear correlation coefficient (i.e., *r* ≤ −0.5 or *r* ≥ 0.5; Fig. 5). Using this criterion, 32 volcanoes show significant decadal trends in SO_2_ emissions (Fig. 5), and although we highlight some potential arc-scale correlations here, further detailed analyses and other measurements are required to evaluate these findings. Trend analysis reveals that most volcanoes in the Vanuatu arc (Ambrym, Aoba and Yasur) show increased degassing in 2005–2015 (Fig. 5), and the only other detectable volcanic SO_2_ source in Vanuatu (Gaua) also shows a positive trend but with a weaker correlation coefficient (r = 0.38; Supplementary Fig. S11). Both Ebulobo and Paluweh (Flores, Indonesia) show significant positive trends (Fig. 5) and are located in the same region of the Sunda arc (Fig. 1). In the Ryukyu Islands and Kyushu regions of Japan, SO_2_ emissions from Satsuma-Iwojima, Sakura-jima, and Aso all show significant positive trends in 2005–2015 (Fig. 5), and the only other detected volcanic SO_2_ source in this region (Suwanose-jima) also shows a positive trend with a lower correlation coefficient (r = 0.39; Supplementary Fig. S9). In addition, there is independent evidence for increased volcanic activity in the Ryukyu Islands and Kyushu region, including a significant eruption at Aso in October 2016, and elevated unrest at Sakura-jima^51^. A recent study^51^ presents geophysical evidence for magma accumulation at Sakura-jima in the 1996–2007 period, with potential for a repeat of its 1914 Plinian eruption in ~25–30 years. The OMI SO_2_ observations show a substantial increase in SO_2_ degassing from Sakura-jima, particularly in 2011–13 (Fig. 5), indicating that the volcano was releasing more gas in this period largely via an increased frequency of vulcanian eruptions^51^. However, since 2013 the SO_2_ emissions from Sakura-jima have declined below the decadal mean (Fig. 5), and so the future evolution of its activity is unclear. Nevertheless, the observed degassing over the past decade may have important implications for future activity at Sakura-jima. For example, the sustained release of SO_2_ could be ‘defusing’ the potential climate impact of a future Plinian eruption, and/or could render a combined explosive-effusive eruption (such as the 1914 event) more likely due to limited gas supply. Gas overpressure and compressibility are rarely factored into models of volcano deformation^52^ and the SO_2_ emissions could also indicate a contribution to the deformation signal due to volatile overpressure in the magma reservoir.

In summary, while the correlated trends in SO_2_ emissions observed in some arcs could be purely coincidental, possible links to underlying regional- or arc-scale geophysical processes (e.g., a coincident pulse in shallow magma supply) merit further investigation but cannot be confirmed on the basis of SO_2_ emissions alone. Regardless of the underlying cause, our trend analysis (Fig. 5) provides new insight into the locations of increased volcanic SO_2_ degassing over the past decade, which would be good targets for increased monitoring (if not already in place), and into volcanoes undergoing long-term decline.

### Pre-eruptive volcanic degassing

Global satellite-based SO_2_ surveillance also offers the potential for detection of pre-eruptive degassing at reawakening volcanoes. As noted above, increased SO_2_ emissions at Aso (Japan) beginning in 2011 (Fig. 5; Supplementary Fig. S10) preceded eruptions in 2014–2016^53^. SO_2_ emissions were detected at Sarychev Peak (Kuril Islands) in 2005–2008 and showed a modest increase prior to its large eruption in June 2009^3^ (Supplementary Fig. S13). At Alu-Dalafilla (Ethiopia), weak but detectable SO_2_ emissions were present in 2005–2007 (Supplementary Fig. S16) prior to an unexpected eruption in November 2008^3^. A shallow (~1 km deep) magma chamber has been identified at Alu-Dalafilla^54^, refilling after the 2008 eruption, which is a likely source of the pre-eruptive SO_2_ emissions. Ground deformation data and the longevity of the magmatic system are consistent with the existence of a relatively thick sill^54^; the persistent low SO_2_ flux detected from 2005–2014 (Supplementary Fig. S16) also supports this, although it is possible that some of the SO_2_ detected by OMI may originate from nearby Erta ‘Ale volcano. Continued analysis of global space-based SO_2_ measurements will thus be valuable for volcanic hazard assessment, particularly at unmonitored volcanoes. Although the low temporal resolution of annual mean SO_2_ emissions precludes timely identification of pre-eruptive unrest (unless it spans several years), one possible approach would be to calculate SO_2_ emissions for all volcanic sources based on a 12-month moving average of satellite SO_2_ measurements (or shorter for stronger sources). This would conserve the sensitivity of the technique to the weak SO_2_ degassing expected in the initial stages of pre-eruptive unrest, whilst permitting more timely identification of increased emissions.

### Missing sources and global volcanic CO_2_ emissions

Inevitably, an undetermined number of weaker SO_2_ sources, populating the tail of global SO_2_ flux distribution (Fig. 4), are missing from the inventory. Continued ground-based SO_2_ measurements at low-flux volcanoes^43^,^55^,^56^ are required to constrain these sources. Such measurements are also needed to improve the relatively poor constraints on the component of global volcanic CO_2_ emissions discharged in volcanic plumes^11^, which requires *in-situ* determination of the CO_2_/SO_2_ ratio in the emissions. As shown by Fig. 4, through coordinated efforts such as the Deep Carbon Observatory (DCO; https://deepcarbon.net/)^57^ significant progress has been made towards improving the spatial coverage of CO_2_/SO_2_ measurements, and around 50% of the detected SO_2_ sources in Table 1 have characterized CO_2_/SO_2_ ratios, including many of the strongest sources (Fig. 4), although the frequency of some measurements remains low. Based on our assessment, particular efforts should be made to pursue further CO_2_/SO_2_ measurements in regions such as Indonesia, Papua New Guinea and Kamchatka, in order to improve constraints on the global volcanic CO_2_ flux.

## Conclusions

We believe that the volcanic SO_2_ emissions inventory described here represents the most accurate assessment of contemporary global volcanic SO_2_ degassing, and we encourage its use by the volcanological and atmospheric science communities as a substitute for existing databases^13^,^31^. Techniques such as this represent a major step forward in monitoring global volcanic degassing and ensure that few, if any, significant sources of volcanic SO_2_ will remain undetected in the future, provided that satellite instruments with comparable sensitivity to OMI continue to be deployed (e.g., the Tropospheric Monitoring Instrument [TROPOMI], scheduled for launch on board the Copernicus Sentinel 5-Precursor satellite in 2017; http://www.tropomi.eu). Efforts to further characterize and validate the derived SO_2_ emissions are strongly encouraged, particularly at those sources with no prior recorded measurements.

We have highlighted several potential applications of the new inventory, including the identification of regional- and arc-scale trends in SO_2_ emissions, and improvement of constraints on global volcanic CO_2_ emissions via measurement of CO_2_/SO_2_ ratios (and their temporal variation) at sources where this information is currently lacking. Ongoing updates to the inventory will potentially provide opportunities to identify pre-eruptive degassing at reawakening volcanoes, and correlate SO_2_ flux data with other geophysical data (e.g., ground deformation measured by InSAR) on a larger scale to elucidate volcanic processes. As a final point, the inventory demonstrates the remarkable persistence of passive volcanic degassing, and as anthropogenic SO_2_ emissions continue to steadily decline, the volcanic contribution to atmospheric sulfur loading will inexorably increase.

## Additional Information

**How to cite this article:** Carn, S. A. *et al*. A decade of global volcanic SO_2_ emissions measured from space. *Sci. Rep.* **7**, 44095; doi: 10.1038/srep44095 (2017).

**Publisher's note:** Springer Nature remains neutral with regard to jurisdictional claims in published maps and institutional affiliations.

## Supplementary Material

Supplementary Information

Supplementary Dataset 1

## Figures and Tables

**Table 1:** Mean SO_2_ fluxes (2005–2015) for sources of passive volcanic SO_2_ degassing detected by OMI.

Rank^1^	Volcano^2^	Country	Mean SO_2_ flux (t/d)	1σ (t/d)	Prev. SO_2_ flux (t/d)^3^
1	Ambrym (1)	Vanuatu	7356	3168	5400
2	Kilauea (3)	USA	5019	2275	1800
3	Bagana (4)	Papua New Guinea	3779	886	2000
4	Nyiragongo + Nyamuragira (2)	Democratic Republic of Congo	3533	2408	2600
5	Aoba (6)	Vanuatu	2870	1229	2200
6	Mt. Etna (9)	Italy	2039	522	1277
7	Tavurvur (82)	Papua New Guinea	1729	2535	110
8	Dukono (10)	Indonesia	1726	611	—
9	Popocatepetl (7)	Mexico	1658	893	2450
10	Manam (12)	Papua New Guinea	1484	753	180
11	Yasur (8)	Vanuatu	1408	563	633
12	Anatahan (83)	Northern Mariana Islands	1335	1867	4367
13	Soufriere Hills (42)	Montserrat (UK)	1296	761	574
14	Nevado del Ruiz (5)	Colombia	1074	1376	1900
15	Sakura-jima (35)	Japan	1056	757	1640
16	Miyake-jima (63)	Japan	1018	934	2120
17	Karymsky (14)	Russia	911	250	75
18	Masaya (18)	Nicaragua	867	364	800
19	Suwanose-jima (17)	Japan	863	314	670
20	Bromo + Semeru (13)	Indonesia	775	298	212
21	Mutnovsky + Gorely (55)	Russia	753	690	1030
22	Turrialba + Poas (16)	Costa Rica	751	681	2754
23	Kizimen (84)	Russia	711	1544	100
24	Avachinsky (26)	Russia	707	619	—
25	Lewotolo + Batu Tara (28)	Indonesia	632	177	—
26	Ijen + Raung (19)	Indonesia	631	238	—
27	Ulawun (24)	Papua New Guinea	630	581	640
28	Langila (48)	Papua New Guinea	629	527	250
29	Aso (11)	Japan	628	492	410
30	Nevado del Huila (44)	Colombia	627	665	—
31	San Cristobal + Telica (25)	Nicaragua	621	283	690
32	Satsuma-Iwojima (20)	Japan	585	190	574
33	Pagan (73)	Northern Mariana Islands	583	547	—
34	Kliuchevskoi + Bezymianny (32)	Russia	580	461	700
35	Shiveluch (31)	Russia	530	284	500
36	Chikurachki + Ebeko (21)	Russia	496	468	100
37	Mayon (60)	Philippines	453	274	530
38	Asama (15)	Japan	449	430	360
39	Gaua (30)	Vanuatu	434	382	2959
40	Sirung (29)	Indonesia	373	162	—
41	Redoubt (85)	USA	368	1051	657
42	Shishaldin (23)	USA	347	278	—
43	Tungurahua (46)	Ecuador	342	235	1460
44	Copahue (22)	Chile	341	425	—
45	Sinabung (36)	Indonesia	327	595	—
46	Karangetang (33)	Indonesia	313	85	—
47	Krakatau (58)	Indonesia	303	252	190
48	Kerinci (45)	Indonesia	294	99	—
49	Tofua (56)	Tonga	284	89	—
50	Villarrica (41)	Chile	281	160	320
51	Michael (43)	South Sandwich Isl. (UK)	263	63	—
52	Sarychev (54)	Russia	260	324	100
53	Tinakula (86)	Solomon Islands	256	276	—
54	Veniaminof (87)	USA	255	214	—
55	White Island (49)	New Zealand	254	107	430
56	Fuego + Pacaya (50)	Guatemala	252	46	280
57	Lastarria (39)	Chile	248	62	884
58	Santa Maria (52)	Guatemala	247	119	120
59	Barren Island (72)	India	243	341	—
60	Ubinas (37)	Peru	222	252	—
61	Galeras (88)	Colombia	218	317	450
62	Bulusan (70)	Philippines	206	199	370
63	Slamet (38)	Indonesia	206	132	58
64	Reventador (57)	Ecuador	206	187	450
65	Lokon-Empung (59)	Indonesia	204	154	—
66	Korovin (65)	USA	198	160	—
67	Kudriavy (74)	Russia	187	103	90
68	Stromboli (53)	Italy	181	82	200
69	Cleveland (77)	USA	152	142	—
70	Montagu (62)	South Sandwich Isl. (UK)	142	179	—
71	Ketoi (79)	Russia	139	151	—
72	Chiginagak (69)	USA	138	127	—
73	Tokachi (61)	Japan	135	98	175
74	Piton de la Fournaise (34)	Reunion Island, France	134	162	—
75	Spurr (64)	USA	106	106	106
76	Jebel at Tair (89)	Yemen	103	295	—
77	Santa Ana (78)	El Salvador	97	180	120
78	San Miguel (51)	El Salvador	88	134	260
79	Sabancaya (27)	Peru	87	158	—
80	Ebulobo (67)	Indonesia	86	63	—
81	Isluga (68)	Chile	78	107	—
82	Rinjani (40)	Indonesia	74	131	—
83	Augustine (75)	USA	73	140	48
84	Sangeang Api (47)	Indonesia	71	150	—
85	Kanlaon (81)	Philippines	70	182	—
86	Alu-Dalafilla + Erta Ale (71)	Ethiopia	64	24	60
87	Paluweh (76)	Indonesia	60	65	—
88	Gareloi (66)	USA	52	47	—
89	Erebus (80)	Antarctica	52	31	74
90	Marapi (90)	Indonesia	34	34	—
91	Merapi (91)	Indonesia	32	51	140
	**TOTAL:**		**62965**		

^1^Rank based on mean SO_2_ flux for 2005–2015.

^2^Number in parentheses indicates SO_2_ flux rank in 2015, the most recent year analyzed.

^3^Previously reported SO_2_ flux, if available. All fluxes are derived from [13] or [31], except data for Etna^58^, Popocatepetl^59^, Anatahan^15^, Bromo-Semeru^41^,^44^, Turrialba^24^, Gaua^36^, Redoubt^60^, Krakatau^42^, Lastarria^55^ and Spurr^61^.
