# Supplementary material for: A decade of global volcanic SO2 emissions measured from space
Source: Sci Rep. 2017 Mar 9;7:44095. doi: 10.1038/srep44095 (PMC5343458; doi:10.1038/srep44095)
Supplement: Supplementary Information [file srep44095-s1.pdf]

Supplementary information

Carn, S. A., Fioletov, V., McLinden, C., Li, C. & Krotkov, N. A.

A decade of global volcanic SO_2 emissions measured from space. *Sci. Rep*.

Supplementary Table 1

The complete volcanic SO_2 emissions inventory is provided as an Excel spreadsheet, including annual SO_2 emissions and 1σ emission uncertainties for each volcanic source, and statistics from a weighted linear regression fit to the annual mean SO_2 emissions.

Supplementary Figures

The following figures show a global map of the volcanic SO_2 sources (Fig. S1), regional maps of some volcanic SO_2 sources (Figs. S2-S8), and time-series plots of annual mean SO_2 fluxes for 2005-2015 for the volcanic sources ranked 11-91 in the volcanic SO_2 emissions inventory (Figs. S9-S16).

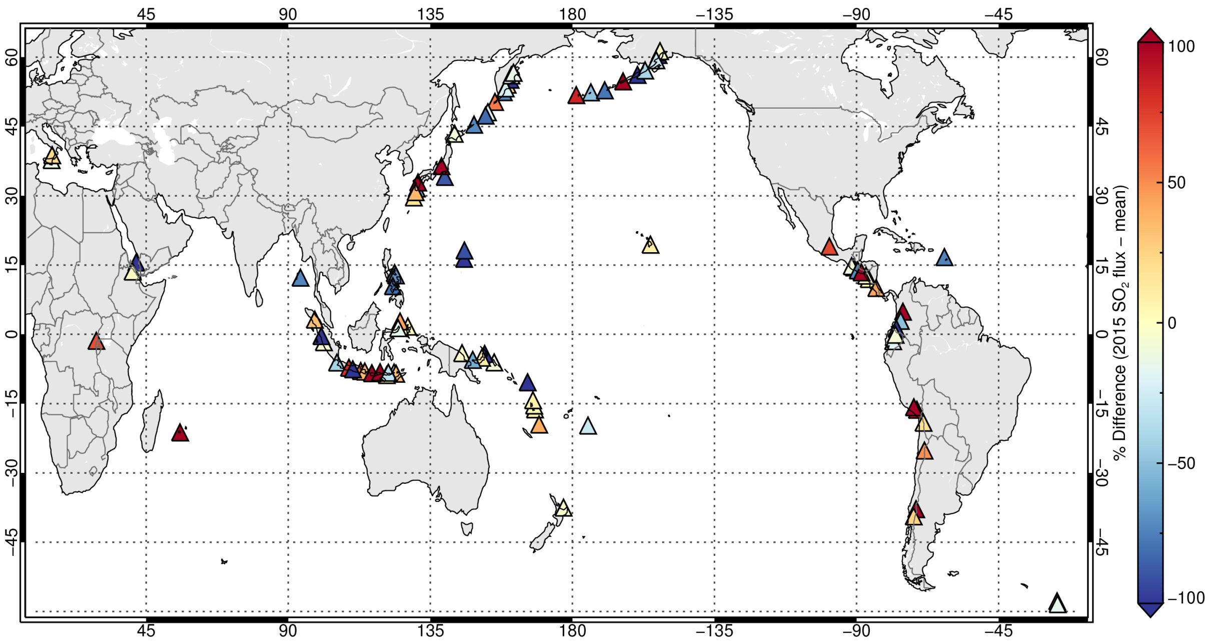

Figure S1. Global map of 91 volcanic SO_2 sources (*triangles*) in the OMI-derived SO_2 emissions inventory. Sources are colored based on the % difference between their annual mean SO_2 emissions in 2015 and their 2005-2015 decadal mean SO_2 flux. Hence, warm and cold colors indicate sources with 2015 SO_2 emissions above and below the long-term average, respectively. More detailed regional maps of the volcanic SO_2 sources are provided in Figures S2-S8. Map generated using Interactive Data Language (IDL) version 8.5.1 (http://www.harrisgeospatial.com/).

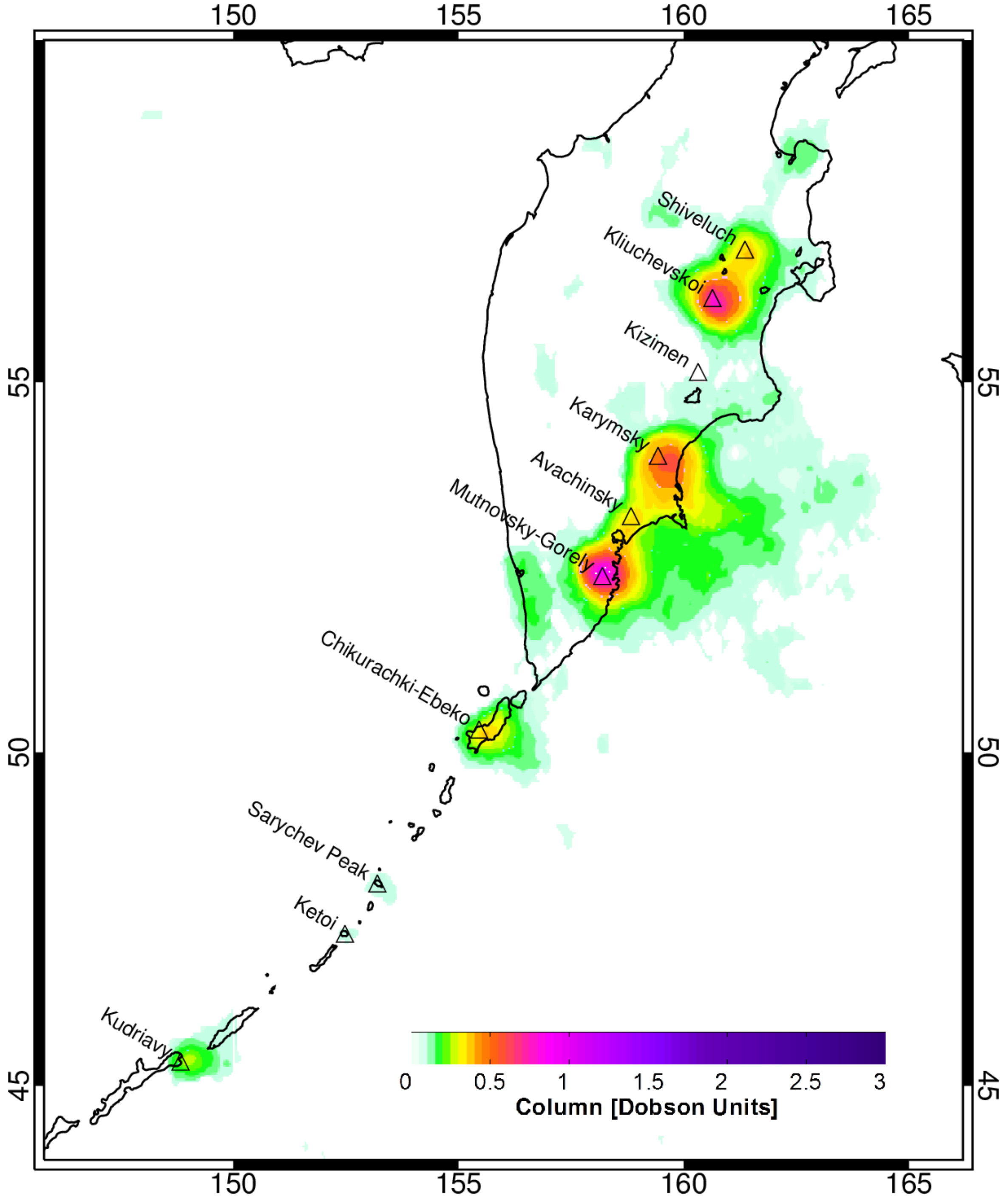

Figure S2. Mean OMI SO_2 columns (in Dobson Units [DU]; 1 DU = 2.69×10^{16} molecules cm^{-2}) for 2005-2007 over Kamchatka and the Kuril Islands (Russia). The volcanic SO_2 sources (including paired sources) responsible for the observed SO_2 emissions are labeled. The absence of an SO_2 anomaly (e.g., at Kizimen) indicates either that the volcano was not active in 2005-2007 or that its emissions were below detection limits. Map generated using IDL version 8.5.1 (http://www.harrisgeospatial.com/).

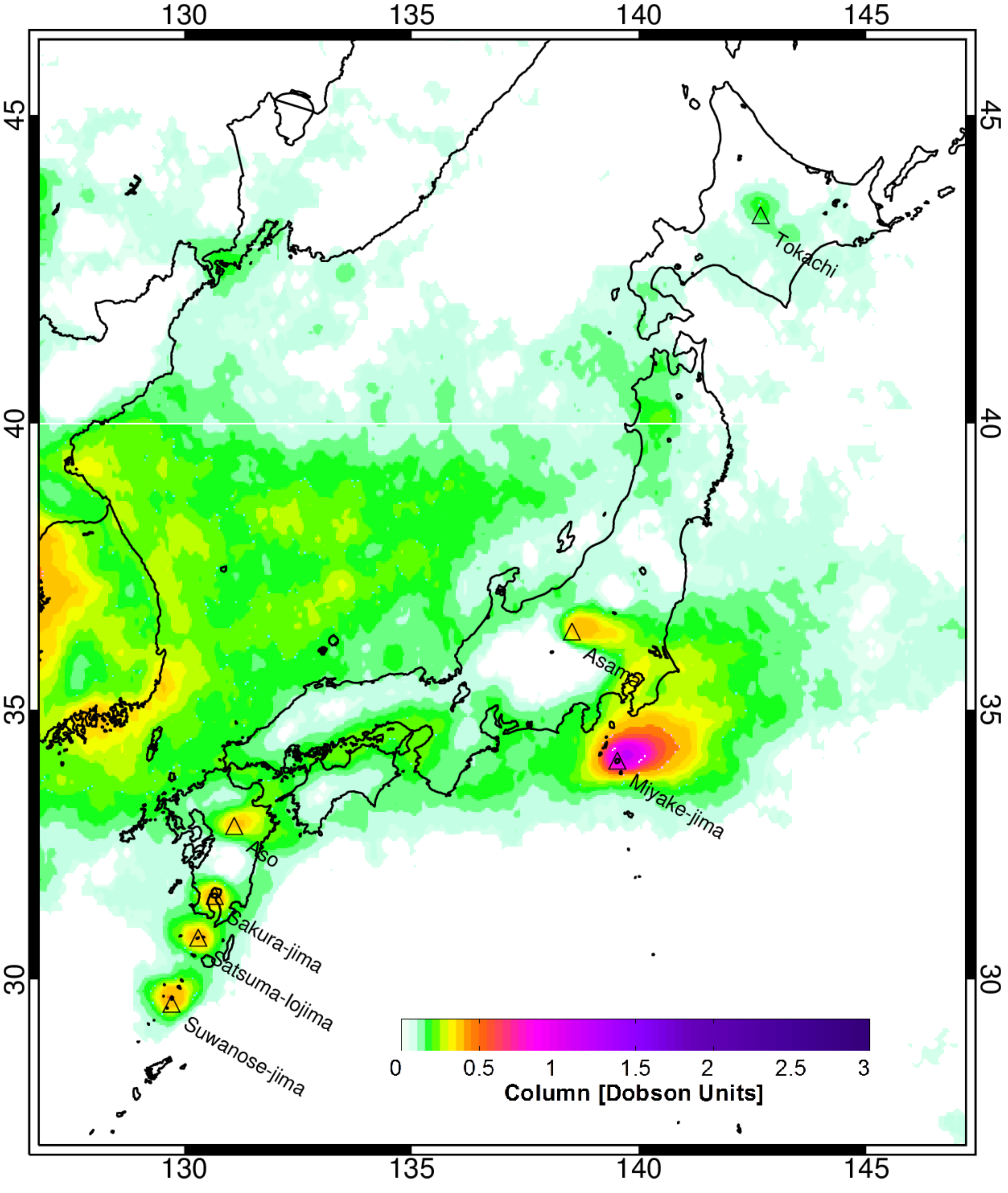

Figure S3. Mean OMI SO_2 columns (DU) for 2005-2007 over Japan. The volcanic SO_2 sources responsible for the observed SO_2 emissions are labeled. Elevated SO_2 columns to the west of Japan are derived from anthropogenic pollution in China. Map generated using IDL version 8.5.1 (http://www.harrisgeospatial.com/).

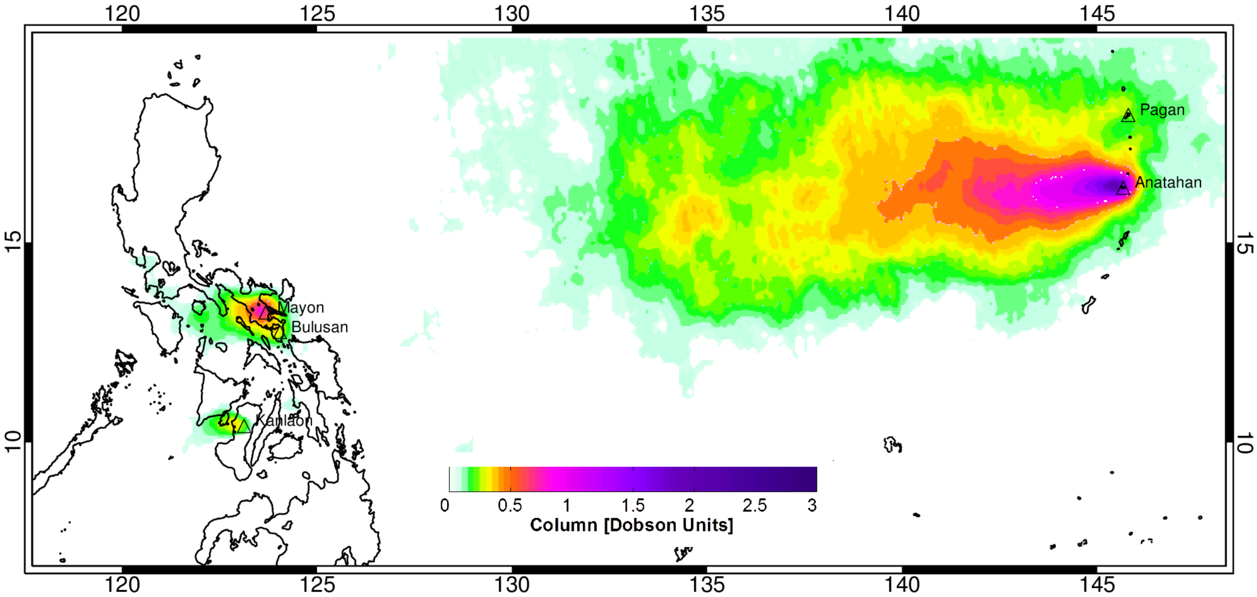

Figure S4. Mean OMI SO_2 columns (DU) for 2005-2007 over the Philippines and the Commonwealth of the Northern Mariana Islands (CNMI, USA). The volcanic SO_2 sources responsible for the observed SO_2 emissions are labeled. Map generated using IDL version 8.5.1 (http://www.harrisgeospatial.com/).

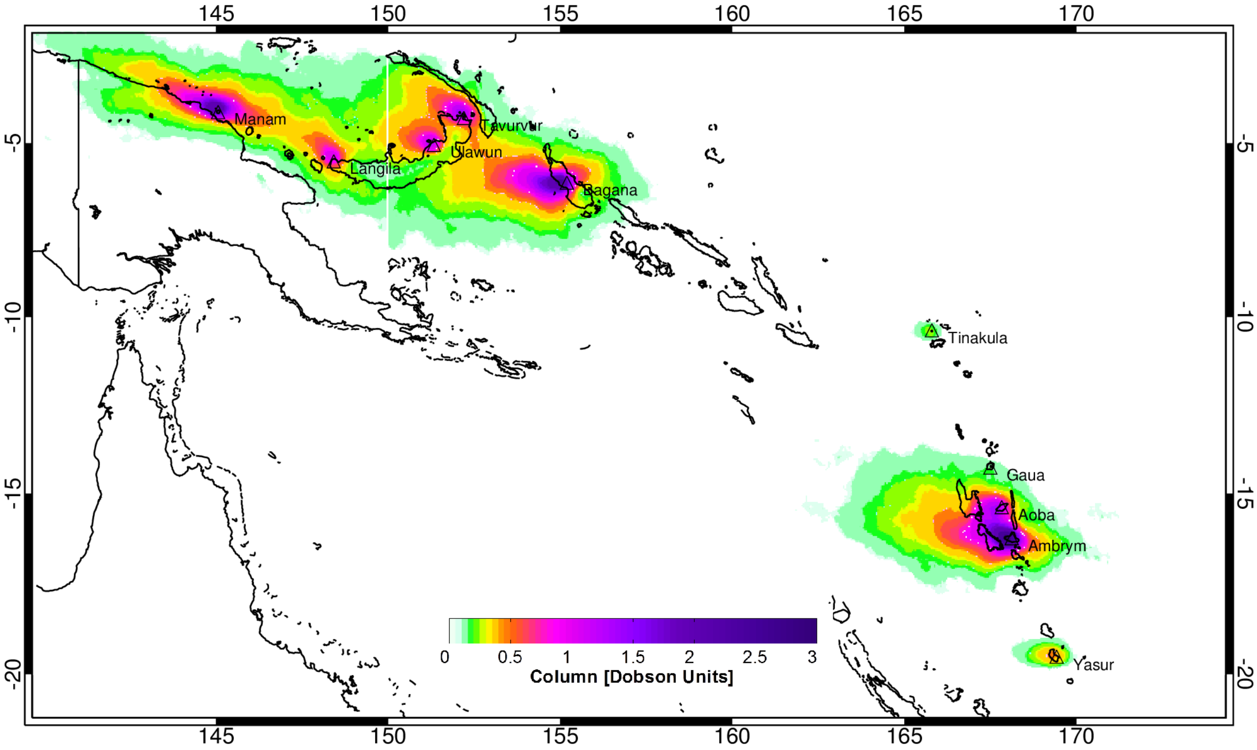

Figure S5. Mean OMI SO_2 columns (DU) for 2005-2007 over Papua New Guinea, the Solomon Islands and Vanuatu. The volcanic SO_2 sources responsible for the observed SO_2 emissions are labeled. Map generated using IDL version 8.5.1 (http://www.harrisgeospatial.com/).

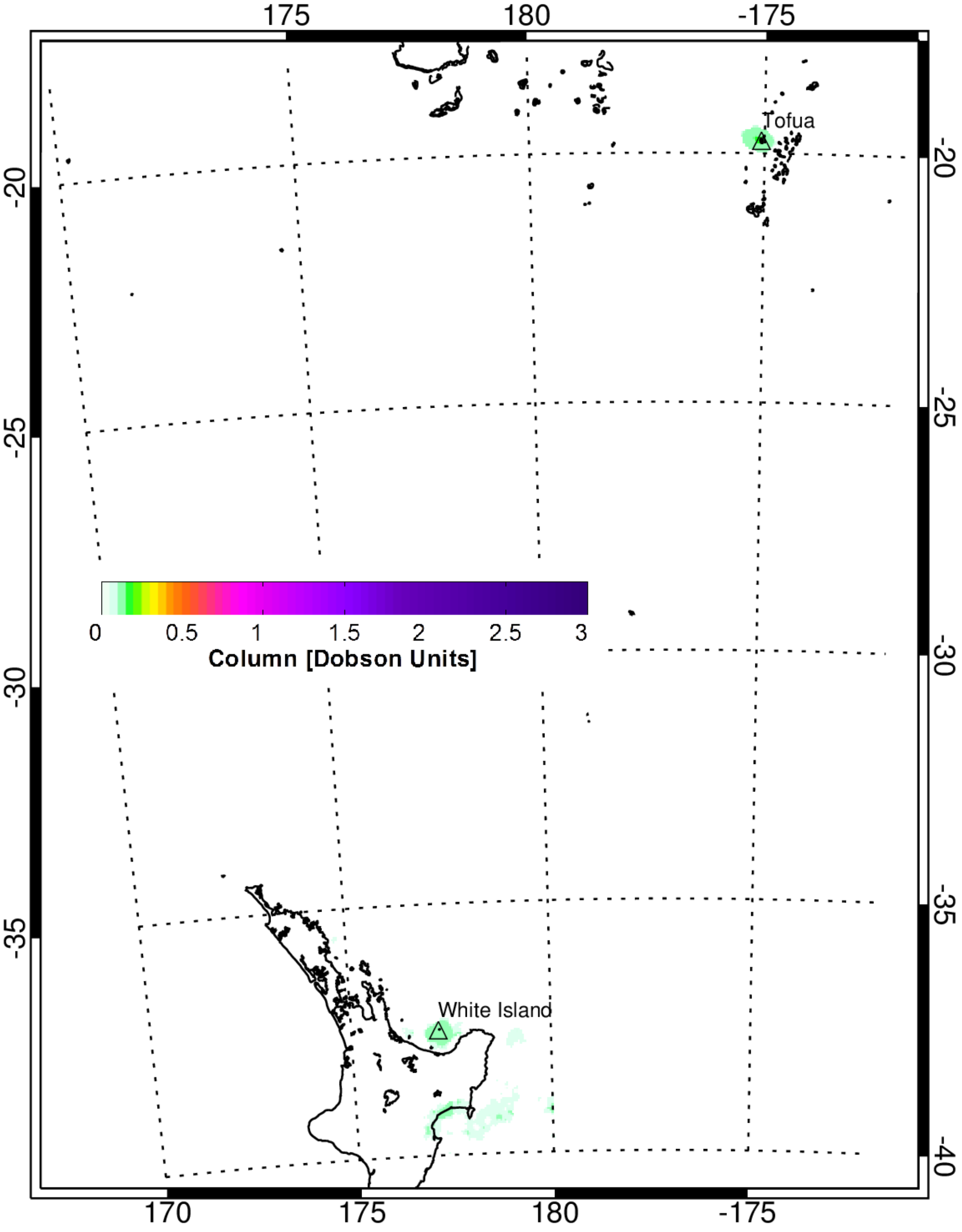

Figure S6. Mean OMI SO_2 columns (DU) for 2005-2007 over New Zealand and Tonga. The volcanic SO_2 sources responsible for the observed SO_2 emissions are labeled. Map generated using IDL version 8.5.1 (http://www.harrisgeospatial.com/).

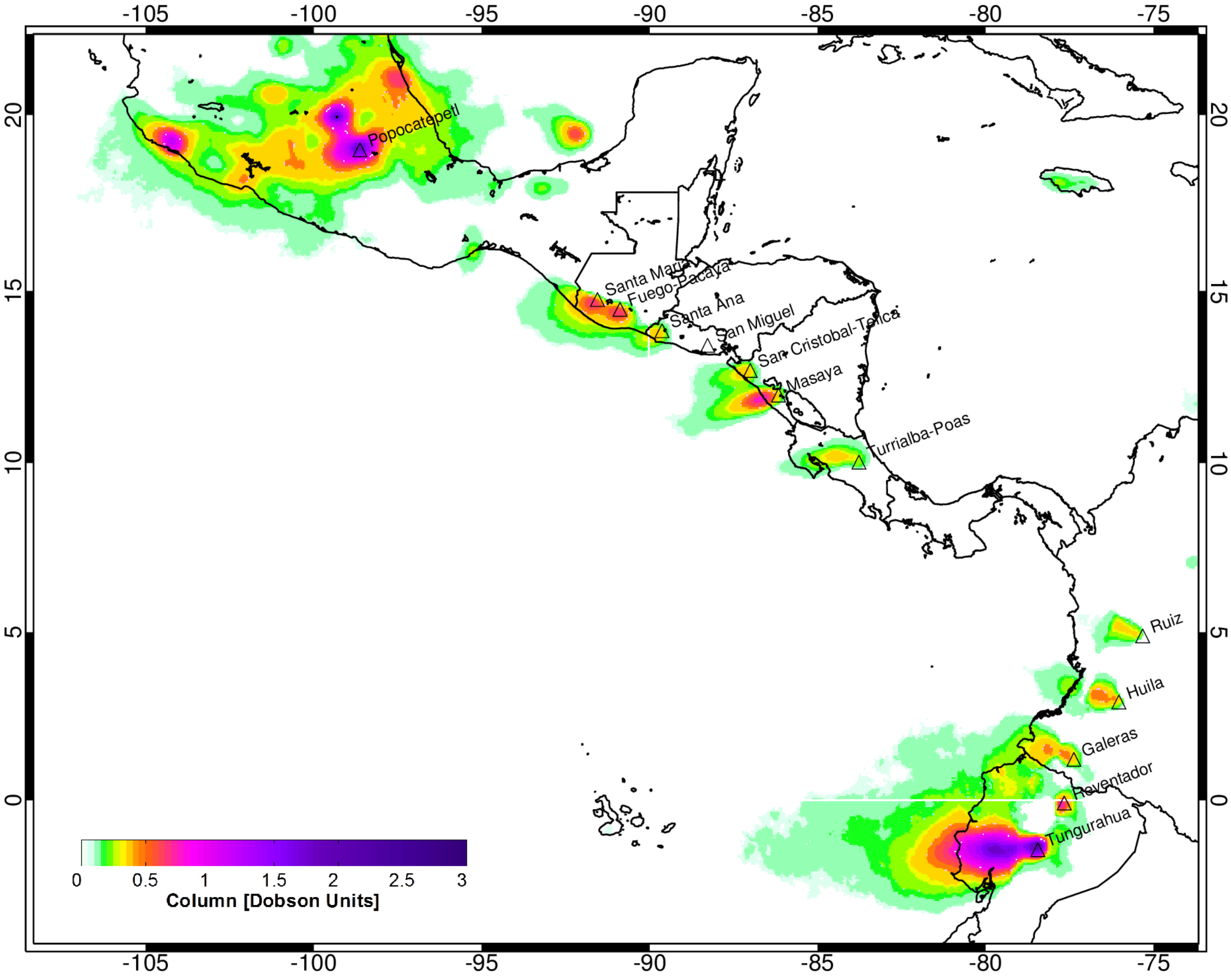

Figure S7. Mean OMI SO_2 columns (DU) for 2005-2007 over Mexico, Guatemala, El Salvador, Nicaragua, Costa Rica, Colombia and Ecuador. The volcanic SO_2 sources (including paired sources) responsible for the observed SO_2 emissions are labeled. Anthropogenic SO_2 sources in Mexico are also apparent. Map generated using IDL version 8.5.1 (http://www.harrisgeospatial.com/).

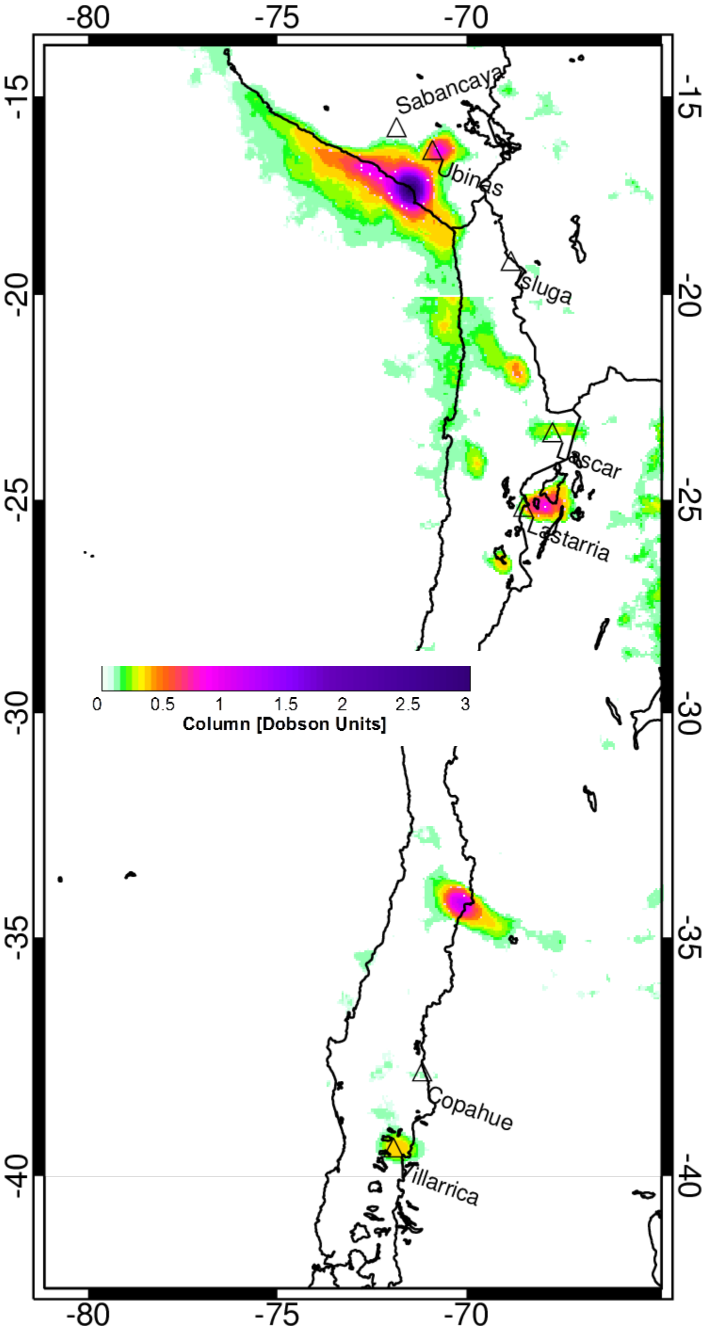

Figure S8. Mean OMI SO_2 columns (DU) for 2005-2007 over Peru, Chile and Argentina. The volcanic SO_2 sources responsible for the observed SO_2 emissions are labeled. Anthropogenic SO_2 sources in Peru and Chile are also apparent. The absence of an SO_2 anomaly (e.g., at Sabancaya) indicates either that the volcano was not active in 2005-2007 or that its emissions were below detection limits. A possible SO_2 signal is associated with Lascar, but this volcano is not included in the emissions inventory. Map generated using IDL version 8.5.1 (http://www.harrisgeospatial.com/).

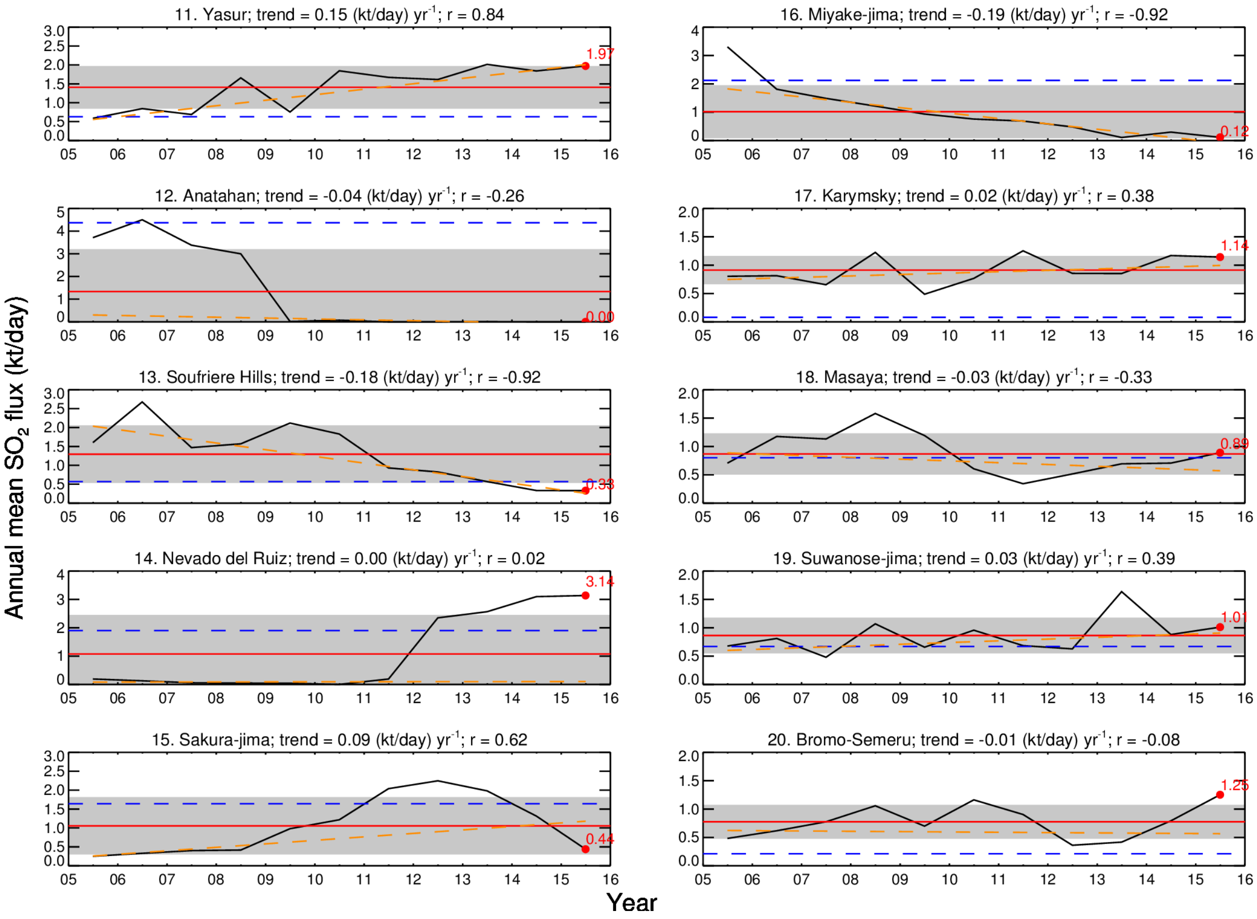

Figure S9. OMI-derived annual mean SO_2 fluxes in 2005-2015 for the volcanic SO_2 sources ranked 11-20 in the inventory. Plots are titled with the volcanic source name and rank, and the trend (slope) and linear correlation coefficient (*r*) of an error-weighted linear regression fit of the annual mean SO_2 fluxes. Each plot shows the annual mean SO_2 fluxes (*solid black line*), mean SO_2 flux in 2015 (*labeled red dot*), linear regression trend line (*dashed orange line*), decadal mean SO_2 flux (*horizontal red line*), ±1 standard deviation of the decadal mean SO_2 flux (*gray band*), and an independent estimate of SO_2 flux (*horizontal dashed blue line*) from a recent compilation[1] or another source. If the latter is not available, the plot is labeled with 'N/A'. Here, independent SO_2 flux data for Anatahan and Bromo-Semeru are from [2] – [4].

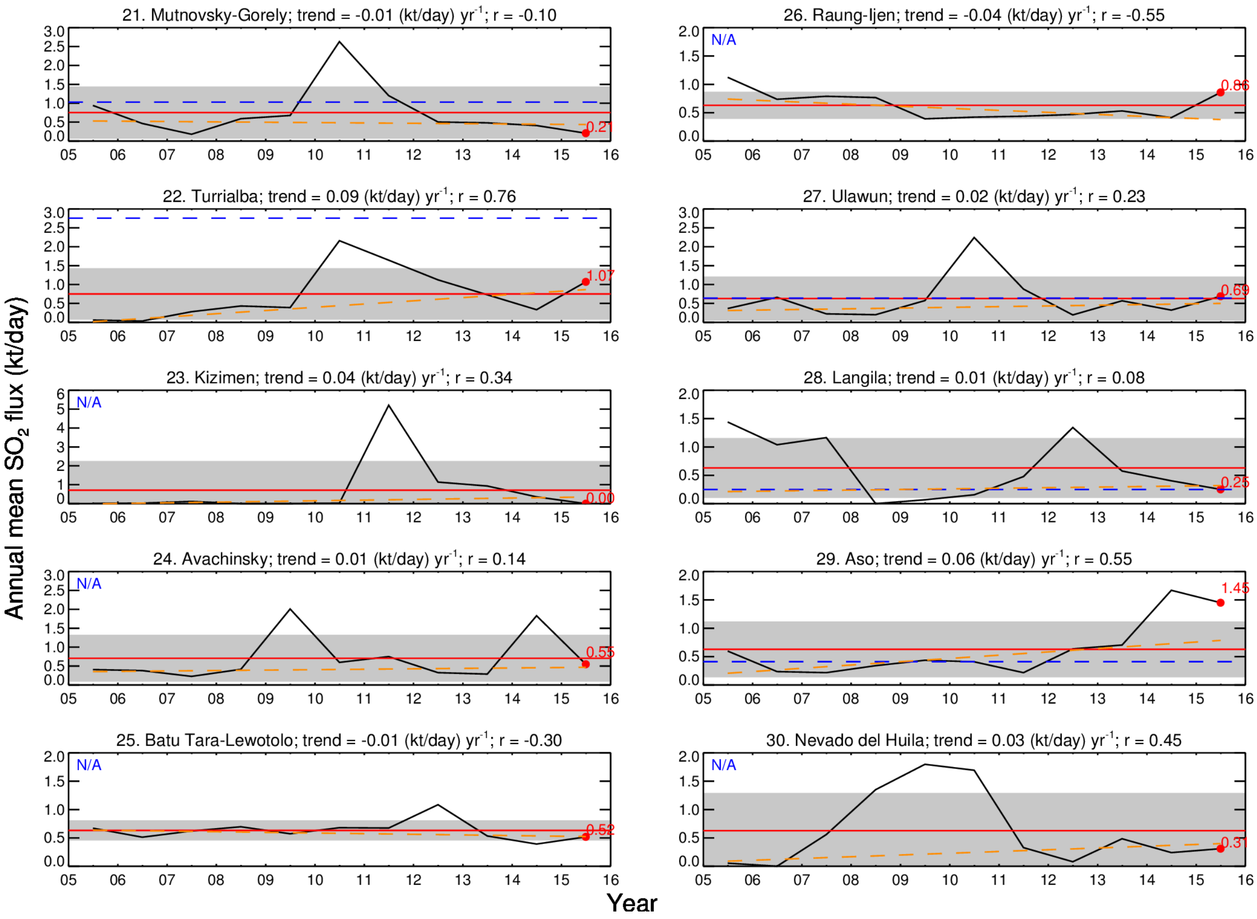

Figure S10. OMI-derived annual mean SO_2 fluxes in 2005-2015 for the volcanic SO_2 sources ranked 21-30 in the inventory. Plots are titled with the volcanic source name and rank, and the trend (slope) and linear correlation coefficient (*r*) of an error-weighted linear regression fit of the annual mean SO_2 fluxes. Each plot shows the annual mean SO_2 fluxes (*solid black line*), mean SO_2 flux in 2015 (*labeled red dot*), linear regression trend line (*dashed orange line*), decadal mean SO_2 flux (*horizontal red line*), ±1 standard deviation of the decadal mean SO_2 flux (*gray band*), and an independent estimate of SO_2 flux (*horizontal dashed blue line*) from a recent compilation[1] or another source. If the latter is not available, the plot is labeled with 'N/A'. Here, independent SO_2 flux data for Turrialba are from [5].

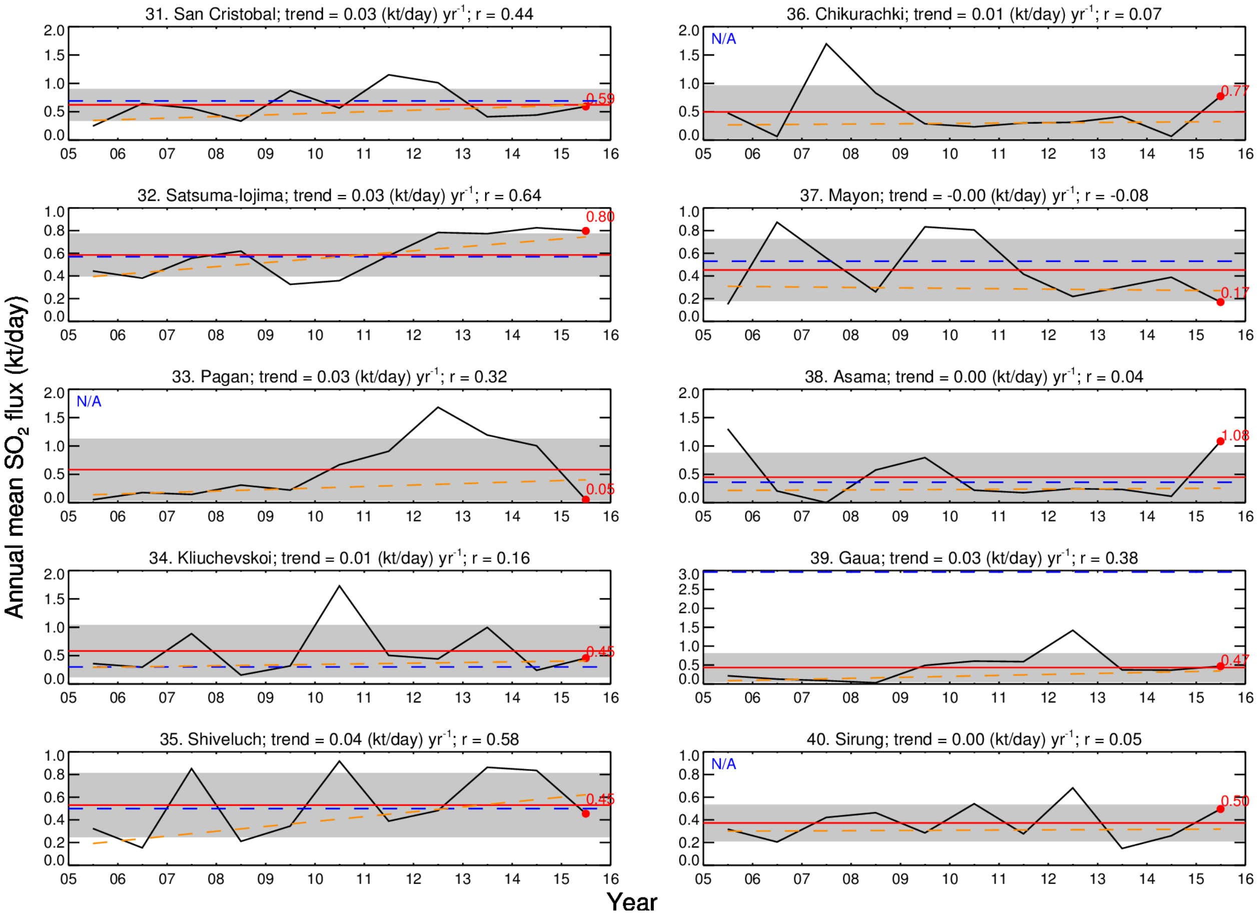

Figure S11. OMI-derived annual mean SO_2 fluxes in 2005-2015 for the volcanic SO_2 sources ranked 31-40 in the inventory. Plots are titled with the volcanic source name and rank, and the trend (slope) and linear correlation coefficient (*r*) of an error-weighted linear regression fit of the annual mean SO_2 fluxes. Each plot shows the annual mean SO_2 fluxes (*solid black line*), mean SO_2 flux in 2015 (*labeled red dot*), linear regression trend line (*dashed orange line*), decadal mean SO_2 flux (*horizontal red line*), ±1 standard deviation of the decadal mean SO_2 flux (*gray band*), and an independent estimate of SO_2 flux (*horizontal dashed blue line*) from a recent compilation[1] or another source. If the latter is not available, the plot is labeled with 'N/A'. Here, independent SO_2 flux data for Gaua are from [6].

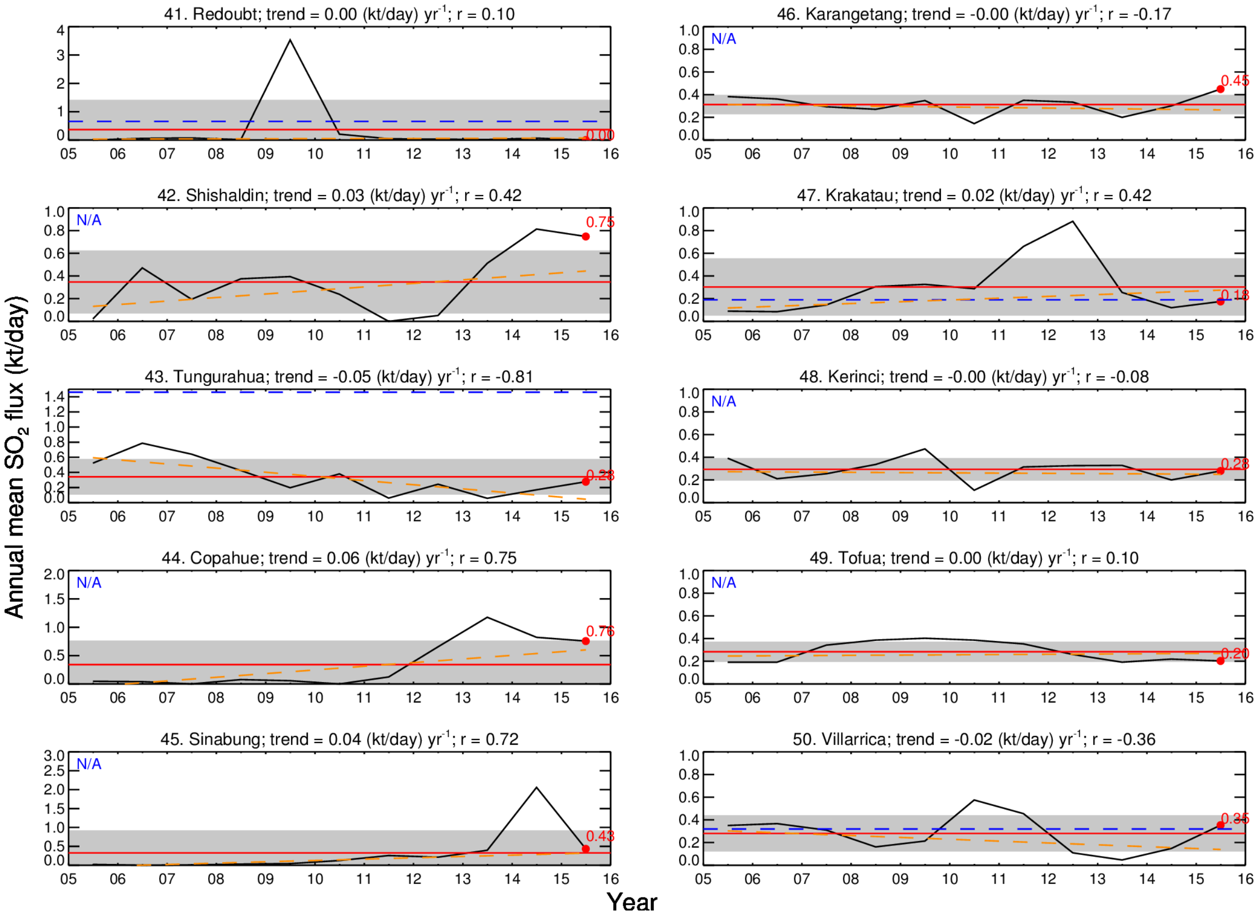

Figure S12. OMI-derived annual mean SO_2 fluxes in 2005-2015 for the volcanic SO_2 sources ranked 41-50 in the inventory. Plots are titled with the volcanic source name and rank, and the trend (slope) and linear correlation coefficient (r) of an error-weighted linear regression fit of the annual mean SO_2 fluxes. Each plot shows the annual mean SO_2 fluxes (*solid black line*), mean SO_2 flux in 2015 (*labeled red dot*), linear regression trend line (*dashed orange line*), decadal mean SO_2 flux (*horizontal red line*), ±1 standard deviation of the decadal mean SO_2 flux (*gray band*), and an independent estimate of SO_2 flux (*horizontal dashed blue line*) from a recent compilation[1] or another source. If the latter is not available, the plot is labeled with 'N/A'. Here, independent SO_2 flux data for Redoubt and Krakatau are from [7] and [8], respectively.

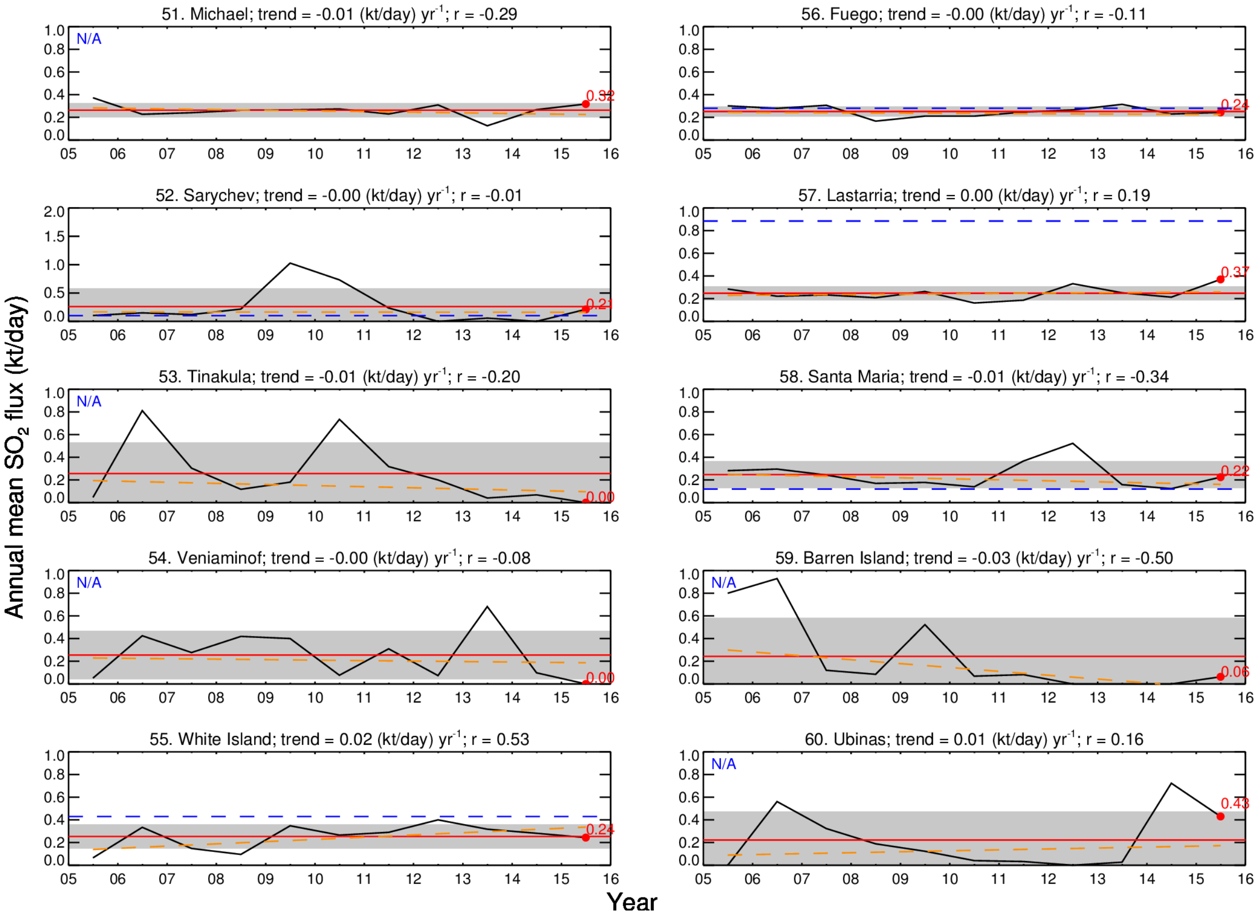

Figure S13. OMI-derived annual mean SO_2 fluxes in 2005-2015 for the volcanic SO_2 sources ranked 51-60 in the inventory. Plots are titled with the volcanic source name and rank, and the trend (slope) and linear correlation coefficient (*r*) of an error-weighted linear regression fit of the annual mean SO_2 fluxes. Each plot shows the annual mean SO_2 fluxes (*solid black line*), mean SO_2 flux in 2015 (*labeled red dot*), linear regression trend line (*dashed orange line*), decadal mean SO_2 flux (*horizontal red line*), ±1 standard deviation of the decadal mean SO_2 flux (*gray band*), and an independent estimate of SO_2 flux (*horizontal dashed blue line*) from a recent compilation[1] or another source. If the latter is not available, the plot is labeled with 'N/A'. Here, independent SO_2 flux data for Lastarria are from [9].

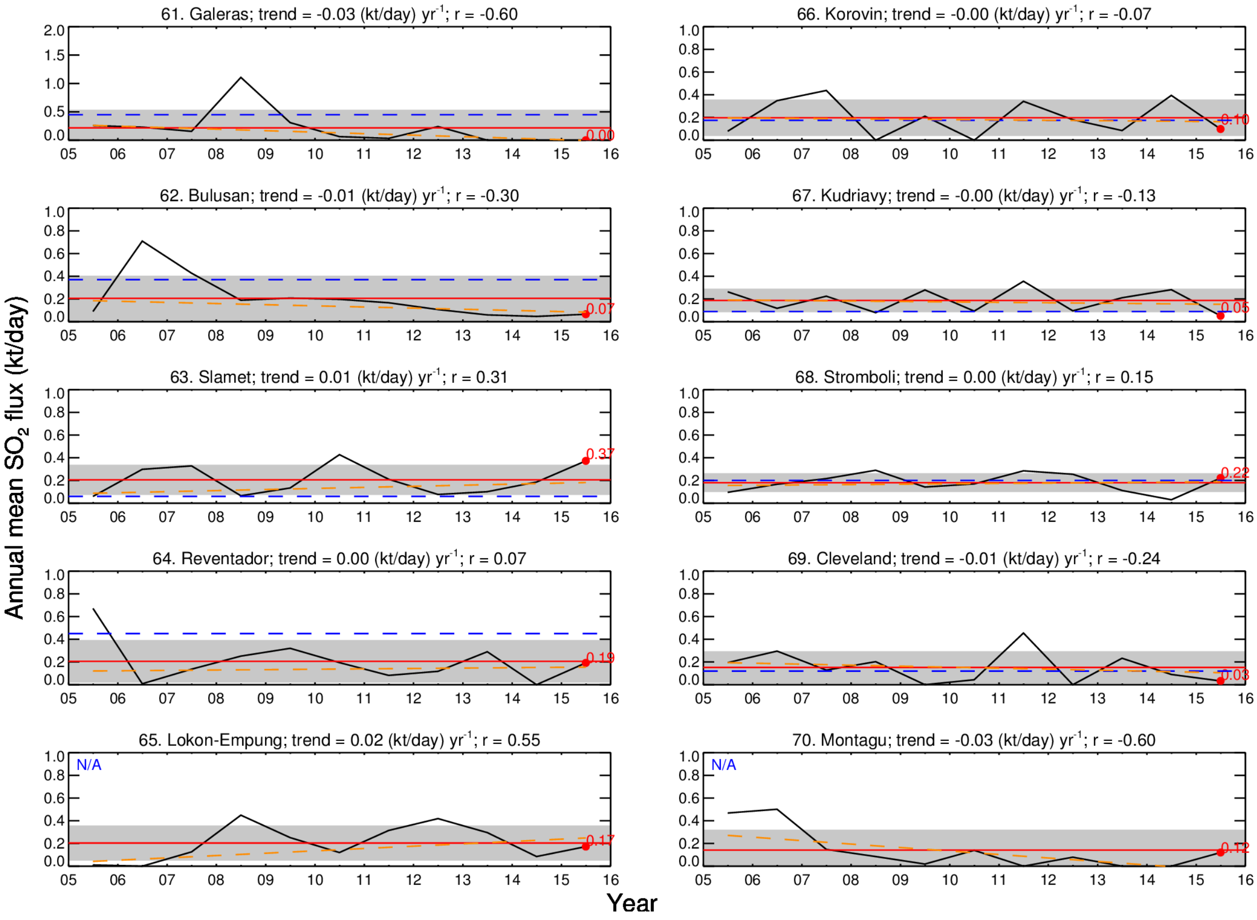

Figure S14. OMI-derived annual mean SO_2 fluxes in 2005-2015 for the volcanic SO_2 sources ranked 61-70 in the inventory. Plots are titled with the volcanic source name and rank, and the trend (slope) and linear correlation coefficient (*r*) of an error-weighted linear regression fit of the annual mean SO_2 fluxes. Each plot shows the annual mean SO_2 fluxes (*solid black line*), mean SO_2 flux in 2015 (*labeled red dot*), linear regression trend line (*dashed orange line*), decadal mean SO_2 flux (*horizontal red line*), ±1 standard deviation of the decadal mean SO_2 flux (*gray band*), and an independent estimate of SO_2 flux (*horizontal dashed blue line*) from a recent compilation[1] or another source. If the latter is not available, the plot is labeled with 'N/A'.

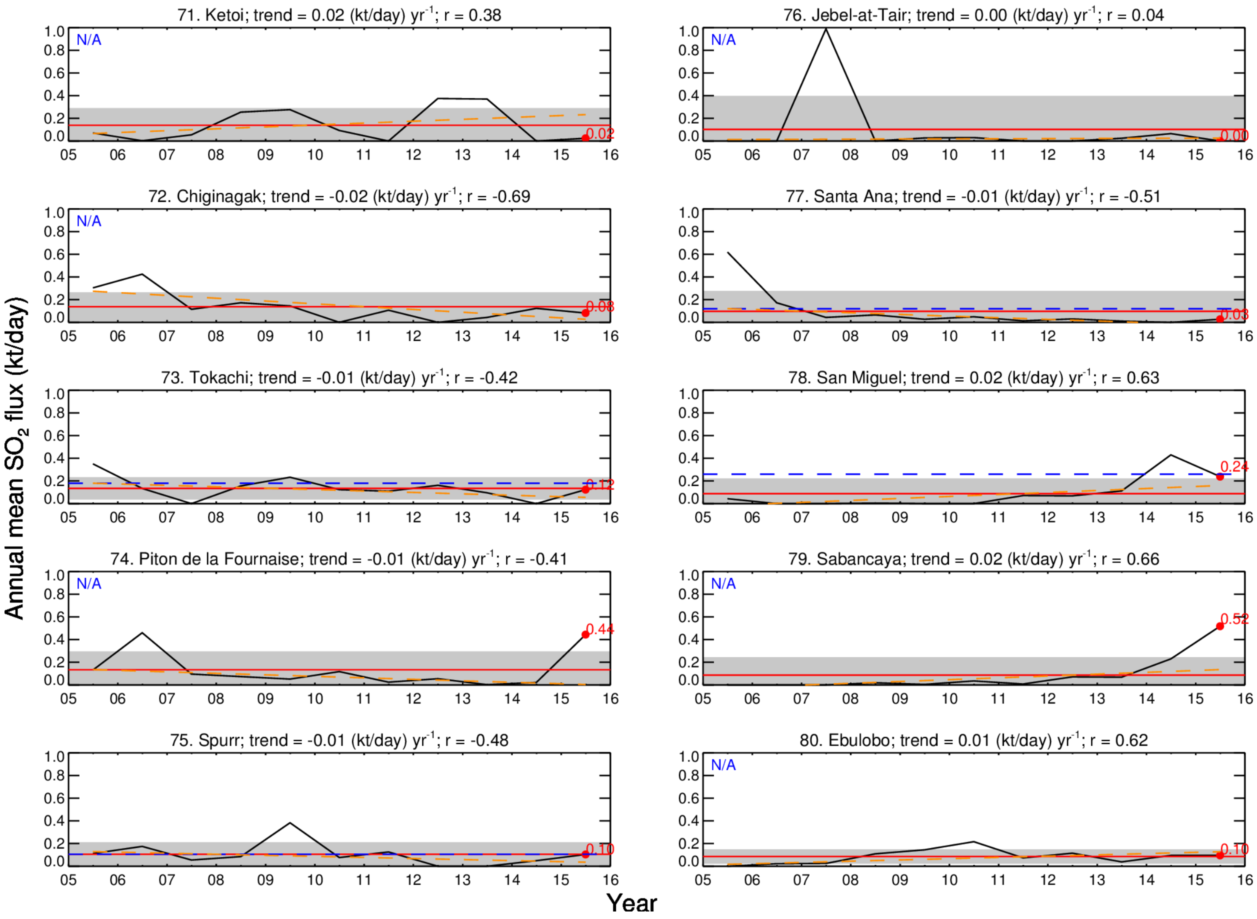

Figure S15. OMI-derived annual mean SO_2 fluxes in 2005-2015 for the volcanic SO_2 sources ranked 71-80 in the inventory. Plots are titled with the volcanic source name and rank, and the trend (slope) and linear correlation coefficient (*r*) of an error-weighted linear regression fit of the annual mean SO_2 fluxes. Each plot shows the annual mean SO_2 fluxes (*solid black line*), mean SO_2 flux in 2015 (*labeled red dot*), linear regression trend line (*dashed orange line*), decadal mean SO_2 flux (*horizontal red line*), ±1 standard deviation of the decadal mean SO_2 flux (*gray band*), and an independent estimate of SO_2 flux (*horizontal dashed blue line*) from a recent compilation[1] or another source. If the latter is not available, the plot is labeled with 'N/A'. Here, independent SO_2 flux data for Spurr are from [10].

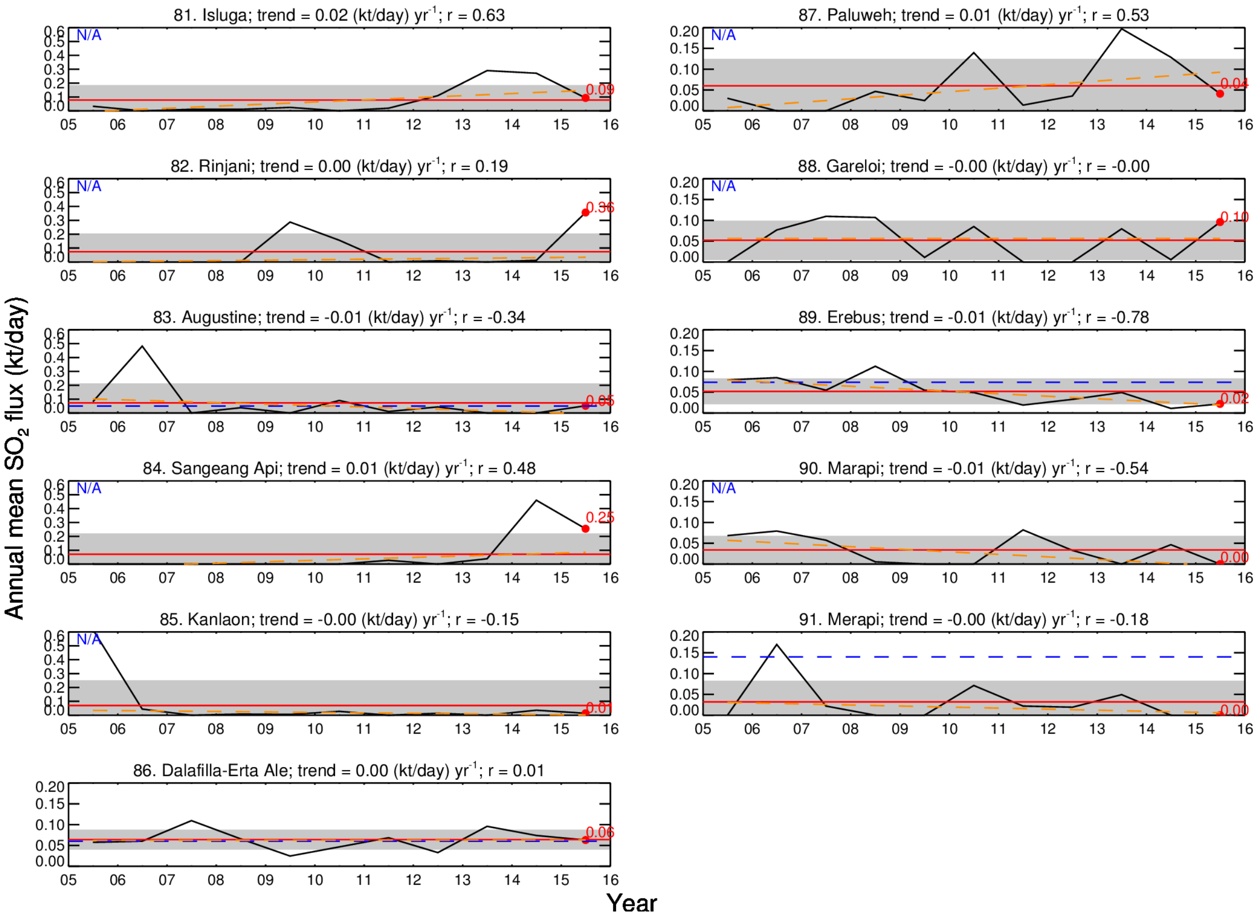

Figure S16. OMI-derived annual mean SO_2 fluxes in 2005-2015 for the volcanic SO_2 sources ranked 81-91 in the inventory. Plots are titled with the volcanic source name and rank, and the trend (slope) and linear correlation coefficient (*r*) of an error-weighted linear regression fit of the annual mean SO_2 fluxes. Each plot shows the annual mean SO_2 fluxes (*solid black line*), mean SO_2 flux in 2015 (*labeled red dot*), linear regression trend line (*dashed orange line*), decadal mean SO_2 flux (*horizontal red line*), ±1 standard deviation of the decadal mean SO_2 flux (*gray band*), and an independent estimate of SO_2 flux (*horizontal dashed blue line*) from a recent compilation[1] or another source. If the latter is not available, the plot is labeled with 'N/A'.

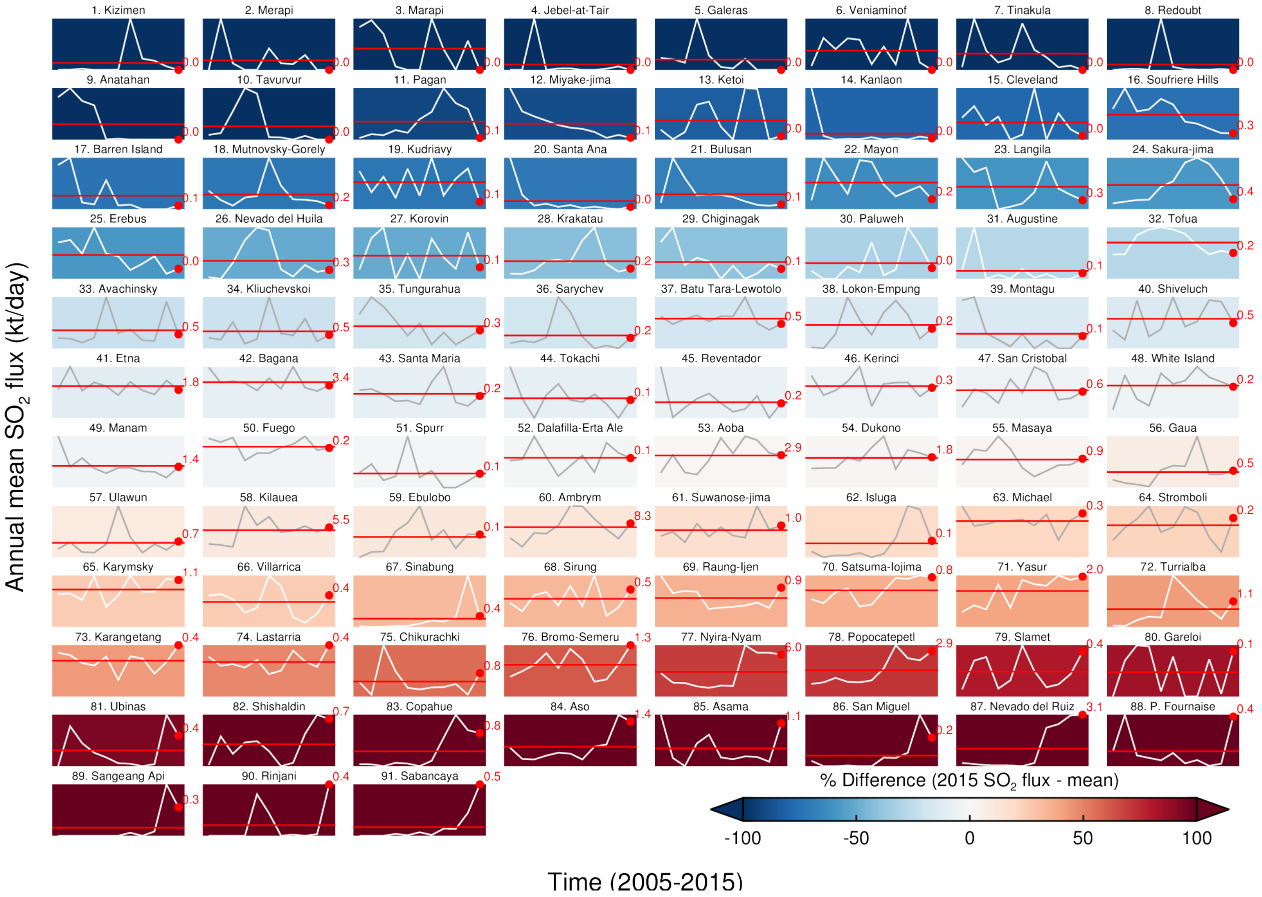

Figure S17. Difference between OMI-derived SO_2 flux in 2015 and the decadal mean flux measured at all detected volcanic SO_2 sources. Plots are ranked in order of % difference between their annual mean SO_2 emissions in 2015 and their 2005-2015 decadal mean SO_2 flux. Hence, warm and cold colors indicate sources with 2015 SO_2 emissions above and below the long-term average, respectively. Each plot shows the annual mean SO_2 fluxes for 2005-2015 (*white-gray line*), the decadal mean SO_2 flux (*red line*) and the annual mean SO_2 flux in 2015 (*labeled red dot*) for each source; axis labels are omitted for clarity. The vertical scale on each plot extends from zero to the maximum measured SO_2 flux.

References

1. Shinohara, H. Volatile flux from subduction zone volcanoes: Insights from a detailed evaluation of the fluxes from volcanoes in Japan. *J. Volcanol. Geotherm. Res*. **268**, 46-63, doi: 10.1016/j.jvolgeores.2013.10.007 (2013).

2. Hilton, D. R., Fischer, T. P., McGonigle, A. J. S. & de Moor, J. M. Variable SO_2 emission rates for Anatahan volcano, the Commonwealth of the Northern Mariana Islands: Implications for deriving arc-wide volatile fluxes from erupting volcanoes. *Geophys. Res. Lett.* **34**, L14315, doi:10.1029/2007GL030405 (2007).

3. Aiuppa, A. *et al.* First determination of magma-derived gas emissions from Bromo volcano, eastern Java (Indonesia). *J. Volcanol. Geotherm. Res*. **304**, 206-213 (2015).

4. Smekens, J.-F., Clarke, A. B., Burton, M. R., Harijoko, A. & Wibowo, H. E. SO_2 emissions at Semeru volcano, Indonesia: Characterization and quantification of persistent and periodic explosive activity. *J. Volcanol. Geotherm. Res*. **300**, 121-128, doi: 10.1016/j.jvolgeores.2015.01.006 (2015).

5. Campion, R. *et al*. Space- and ground-based measurements of sulfur dioxide emissions from Turrialba volcano (Costa Rica). *Bull. Volcanol*. **74**(7), 1757–1770 (2012).

6. Bani, P. *et al.* First arc-scale volcanic SO_2 budget for the Vanuatu archipelago. *J. Volcanol. Geotherm. Res*. **211-212**, 36–46, http://dx.doi.org/10.1016/j.jvolgeores.2011.10.005 (2012).

7. Werner, C. A. *et al.* Degassing of CO_2, SO_2, and H_2S associated with the 2009 eruption of Redoubt Volcano, Alaska, 1989–2006. *J. Volcanol. Geotherm. Res*. **259**, 270-284 (2013).

8. Bani, P. *et al.* First measurement of the volcanic gas output from Anak Krakatau, Indonesia. *J. Volcanol. Geotherm. Res*. **302**, 237-241, doi: 10.1016/j.jvolgeores.2015.07.008 (2015).

9. Tamburello, G., Hansteen, T. H., Bredemeyer, S., Aiuppa, A. & Tassi, F. Gas emissions from five volcanoes in northern Chile and implications for the volatiles budget of the Central Volcanic Zone. *Geophys. Res. Lett*. **41**, 4961–4969, doi:10.1002/2014GL060653 (2014).

10. Werner, C. A., Doukas, M. P. & Kelly, P. J. Gas emissions from failed and actual eruptions from Cook Inlet Volcanoes, Alaska, 1989–2006. *Bull. Volcanol*. **73**(2), 155-173 (2011).
